# The genotypic and phenotypic impact of hypoxia microenvironment on glioblastoma cell lines

**DOI:** 10.1186/s12885-021-08978-z

**Published:** 2021-11-19

**Authors:** Lucy Wanjiku Macharia, Wanjiru Muriithi, Carlos Pilotto Heming, Dennis Kirii Nyaga, Veronica Aran, Marianne Wanjiru Mureithi, Valeria Pereira Ferrer, Attilio Pane, Paulo Niemeyer Filho, Vivaldo Moura-Neto

**Affiliations:** 1 Programa de Pós-Graduação em Anatomia Patológica, Faculdade de Medicina da Universidade Federal do Rio de Janeiro – (PPGAP-UFRJ), Rio de Janeiro, Brazil; 2 Laboratório de Biomedicina do Cérebro- Instituto Estadual do Cérebro Paulo Niemeyer (IECPN), Rio de Janeiro, Brasil. Rua do Rezende, 156 - Centro, Rio de Janeiro, RJ 20231-092 Brasil; 3 Instituto de Ciências Biomédicas da Universidade Federal do Rio de Janeiro (ICB-UFRJ), Rio de Janeiro, Brazil; 4 Faculdade de Medicina da Universidade Federal do Rio de Janeiro, Rio de Janeiro, Brazil; 5 KAVI Institute of Clinical Research, Faculty of Medicine, University of Nairobi, Nairobi, Kenya

**Keywords:** Glioblastoma, Tumorigenesis, hypoxia microenvironment, miRNAs, Stem-like state

## Abstract

**Background:**

Glioblastoma is a fatal brain tumour with a poor patient survival outcome. Hypoxia has been shown to reprogram cells towards a stem cell phenotype associated with self-renewal and drug resistance properties. Activation of hypoxia-inducible factors (HIFs) helps in cellular adaptation mechanisms under hypoxia. Similarly, miRNAs are known to be dysregulated in GBM have been shown to act as critical mediators of the hypoxic response and to regulate key processes involved in tumorigenesis.

**Methods:**

Glioblastoma (GBM) cells were exposed to oxygen deprivation to mimic a tumour microenvironment and different cell aspects were analysed such as morphological changes and gene expression of miRNAs and survival genes known to be associated with tumorigenesis.

**Results:**

It was observed that miR-128a-3p, miR-34-5p, miR-181a/b/c, were down-regulated in 6 GBM cell lines while miR-17-5p and miR-221-3p were upregulated when compared to a non-GBM control. When the same GBM cell lines were cultured under hypoxic microenvironment, a further 4–10-fold downregulation was observed for miR-34-5p, miR-128a-3p and 181a/b/c while a 3–6-fold upregulation was observed for miR-221-3p and 17-5p for most of the cells. Furthermore, there was an increased expression of SOX2 and Oct4, GLUT-1, VEGF, Bcl-2 and survivin, which are associated with a stem-like state, increased metabolism, altered angiogenesis and apoptotic escape, respectively.

**Conclusion:**

This study shows that by mimicking a tumour microenvironment, miRNAs are dysregulated, stemness factors are induced and alteration of the survival genes necessary for the cells to adapt to the micro-environmental factors occurs. Collectively, these results might contribute to GBM aggressiveness.

**Supplementary Information:**

The online version contains supplementary material available at 10.1186/s12885-021-08978-z.

## Background

Glioblastoma (GBM), a grade IV astrocytoma is the most common and fatal type of primary adult brain tumour [1, 2]. Dismal overall prognosis remains for patients with GBM due to a local recurrence despite the concomitant treatment based on chemotherapy using temozolomide (TMZ), radiotherapy and surgery [3, 4]. With treatment, the median survival of patients with GBM is approximately 14.6 months, and their 5-year survival rate is 4–5% [1, 3, 5]. In the tumour mass, only a small fraction of cells known as glioblastoma stem cells (GSCs), is capable of initiating a new tumour due to their self-renewal capability and their resistance to chemo-radiotherapy [6, 7].

Hypoxia is a fundamentally important hallmark of glioblastomas relative to lower-grade astrocytoma and is associated with tumour progression and a poor patient prognosis [8–10]. Pathologically, the hypoxia phenomenon is commonly represented in GBM by a distinctive feature including necrotic foci with surrounding cellular pseudopalisades and microvascular hyperplasia [10, 11]. GBMs are often highly vascularized, and their increased cell proliferation/growth is linked to erratic tumour neovascularization. However, the vasculature is poorly organized and exhibits severe structural and functional abnormalities. Consequently, this leads to regions of the tumour experiencing a reduced supply of oxygen, known as hypoxia [10, 12]. Hypoxia triggers cellular adaptation, drives the selection of more aggressive tumour cells as well as the potentiation of the infiltration and migration of tumour cells [13, 14]. Similarly, the hypoxia microenvironment has been shown to maintain GSCs and promote reprogramming towards a cancer stem–like cell phenotype [15]. To cope with low oxygen availability, intracellular modifications occur in the cells including reprogramming of the metabolic and bioenergetic demands primarily through a number of oxygen-sensing pathways including the hypoxia-inducible factors (HIFs) family of transcription factors -dependent and -independent responses [16]. HIF comprise an oxygen-sensitive alpha subunit and a beta subunit. Under hypoxia, the alpha subunit is stabilized and translocated from the cytoplasm to the nucleus where it heterodimerizes with the constitutively expressed beta subunit [16, 17]. HIF leads to the transcription of genes that regulate processes such as angiogenesis, glycolysis and invasion [8].

Among the non-coding RNAs, microRNAs (miRNAs) are the commonly studied RNAs [18] with a potential capacity of regulating up to 60% of the protein-coding genes [19]. Dysregulated expression of miRNAs has been observed in GBM making them a promising diagnostic biomarker [20]. Seven of the miRNAs used in this study were selected from previous studies as either upregulated or downregulated in glioblastoma samples. Studies have found miR-34 to be downregulated in GBM tissues as compared to healthy controls and to be a key regulator of tumour suppression [21, 22]. The highly conserved miR-34a has been reported to have multiple targets including Notch-1, Notch-2, CDK6, CCND1, MYC, BCL-2, c-Met [21, 23–25] and to be a transcriptional target of p53 [26, 27]. The miRNA 128-3p is a brain enriched miRNA, [28] that also appears to act as a tumour suppressor in glioblastoma whose expression is found reduced in human glioblastoma samples compared to controls devoid of the tumour, also correlating with aggressive human glioma subtypes [29–32]. The miR-128 has been shown to have various targets like PDGFRA, EGFR [33] WEE1, E2F3a, [34, 35] ANGPTL6, Bmi-1 [29] and SUZ12 [36]. The miR-181 family are well known brain-enriched miRNAs [32] found downregulated in all grades of glioma samples compared to normal brain tissues [30, 31, 37] and predicts response to concomitant chemoradiotherapy with temozolomide in glioblastoma patients [38, 39]. The miR-181a can target Bcl-2 [40, 41] while miR-181b and miR-181c can modulate the expression of EGFR [42] and the NOTCH2 pathway [43] respectively. On the other hand, miR-221 has been found upregulated in GBM versus normal brain tissue [30, 31]. miR221 can regulate the STAT3/Akt pathway, the tumour suppressor p27^Kip1^ in glioma cells [44, 45] and can interfere with the p53/Bcl-2/PUMA, p27Kip1 and TGF-*β* signalling [46, 47]. Finally, The miR-17-92 is one of the best-characterized oncogenic miRNAs, a polycistronic miRNA cluster, designated as OncomiR-1 [48]. miR-17 is expressed at higher levels in glioma tissues than in normal brain tissues and is associated with poor patient prognosis [49] targeting PTEN and ATG7 [50, 51]. Additionally, a proto-oncogene, c-Myc [52–54] alongside the Notch and Sonic Hedgehog oncogenic signalling pathways [55] have been found to activate the transcription of the miR-17 family. An intertwined role between microRNAs, hypoxia and the stem cell state has been well explained in our previous work [56].

Oxygen concentration in the human brain ranges between approximately 4.6% O_2_ in the healthy brain to 1.7% O_2_ in a brain with a tumour [57]. GBM cell lines are traditionally cultured in vitro with an O_2_ tension of 20.9%. These oxygen values are far from the experimental in vitro conditions which mean that cell culture is performed in hyperoxic rather than physoxic conditions of respective organs or the physiological oxygen tension reflected in the tumour microenvironment [58, 59]. By growing our cells in a hypoxia microenvironment, we mimicked a glioblastoma hypoxic niche allowing for a more in-depth study of the cellular adaptations. In the present study, we were able to shed light on the morphological changes, miRNAs alterations and pro-survival genes in response to hypoxia (1% O_2_) reflective of possibly the true events that occur in an actual tumour microenvironment. The importance of our work relies on the fact that the miRNA expression profile has not been previously established using our GBM cell lines. Similarly, even though there are advances in the field of tumour hypoxia, in vitro experiments continue to be conducted mainly under normoxic microenvironments that may have influenced previously reported results. The evidence provided by the present study may help to support the development of future effective therapies and the discovery of new therapeutic targets.

## Methods

### Reagents

All components used for the culture media, DMEM/F12 Dulbecco’s Modified Eagle Medium/Nutrient Mixture were supplied by Gibco®; HEPES was supplied by Life Technologies (São Paulo, Brazil) and fetal bovine serum (FBS) was supplied by Invitrogen (Paisley, UK). Penicillin and Streptomycin were purchased from Gibco. Fungizone was purchased from Bristol-Myers Squibb (Princeton, NJ). Glucose was purchased from Merck (Darmstadt, Germany). All culture plates and flasks were obtained from TPP (Trasadingen, Switzerland). Rabbit anti–glial fibrillary acidic protein (#Z0334) and mouse anti-vimentin (#M0725), antibodies were purchased from Dako (Glostrup, Denmark). Protease and phosphatase inhibitors were supplied by Roche (Indianapolis, IN). Antibodies for Oct-4A (#2840) and SOX2 (#D6D9) were purchased from Cell Signaling Technology (Beverly, MA**).** The anti-GLUT-1(#07–1401) and anti-HIF-1α (MAB5382) antibody were purchased from Millipore Corporation (Single Oak drive, CA, USA) Anti-VEGFA (#ab46154) and anti-Survivin (#ab76424) anti-BCL2 (#ab33862) antibodies were purchased from Abcam (Cambridge, MA, USA). The secondary antibodies, conjugated to Alexa Fluor 488(#A-11008, A-21131) and 546 (#A-11010, A-21124) or HRP conjugated anti-mouse (#G-21040) or rabbit (#G-21234) antibodies were purchased from Thermo Scientific (Rockford, USA). PVDF membranes were purchased from Millipore (Billerica, MA). The 4′, 6-diamidino-2-phenylindole (DAPI) was obtained from Sigma (MA, USA). 2× Laemmli buffer and β-mercaptoethanol were purchased from Bio-Rad (São Paulo, Brazil).

### Cells and culture conditions

The GBM02, GBM03, GBM11 and GBM95 glioblastoma cell lines were established and characterized in our laboratory “*Laboratório de Morfogênese Celular –ICB-UFRJ*”, as previously described by Faria et al., [60], while U87 and T98G are ATCC cell lines. The GBM cells were maintained in Dulbecco’s modified Eagle’s medium (DMEM/F12) supplemented with 10% fetal bovine serum (FBS) and maintained in normoxia chamber set at 37 °C in an atmosphere containing 95% air and 5% CO_2_ for the normoxic culture and at 1% O_2_ in a hypoxia chamber (Thermo scientific) for the hypoxia culture for 72 h. A non-GBM control (human astrocyte cell line) was used as control for the miRNA experiments. The H2 human astrocyte cell line was kindly provided by Dr. Luciana Romão from the Institute of Biomedical Sciences - Federal University of Rio de Janeiro (ICB-UFRJ) and was maintained in the same culture conditions as the GBM cell lines.

### Western blotting

GBM cells were cultured in 75 cm^3^ Petri dishes and incubated for 72 h in either normoxic or hypoxic conditions. When the cells were ready, the media was poured out and the Petri dishes were rinsed three times with cold 1X PBS. The Petri dishes were then maintained in ice after which a lysis buffer (1X RIPA (RIPA-25 mM Tris•HCl, pH 7.6, 150 mM NaCl, 1% NP-40, 1% sodium deoxycholate and 0.1% sodium dodecyl sulfate (SDS), supplemented with protease inhibitor was added and then scraped off using a cell scraper. The lysates were sonicated and then centrifuged at 4 °C, 10.000 g for 15 min. The supernatants were analysed for protein content using the Bradford method [61] according to the manufacturer’s instructions. Samples were mixed with sample buffer (2x Laemmli sample buffer and 2-Mercaptoethanol) in a ratio of 1:1 and heated for 5 min at 95 °C. For the immunodetection of proteins, 30-50 μg of total cell proteins were separated by electrophoresis on 10% SDS polyacrylamide gels and transferred to polyvinylidene difluoride (PVDF) membranes. The PVDF membranes were then blocked with 5% non-fat milk in Tris-buffered saline with 0.1% Tween-20 (TBS-T) for 1 h, incubated with specific primary antibodies overnight at 4 °C, washed with TBS-T five times for five minutes each before incubation with horseradish peroxidase-conjugated antibodies. The signals of anti-SOX2/OCT4A (Cell Signaling Technology, 1:1000), anti-Glut-1 (Millipore, 1:500) anti-VEGF (Abcam,1:1000), anti- Survivin (Abcam 1:1000), anti-GFAP (DAKO, 1:1000), anti-Vimentin (Dako, 1:1000). Super-signal West Pico chemiluminescent substrate (Thermo Scientific) and Super-signal West Femto maximum sensitivity substrate (Thermo Scientific) was used to reveal the bands. Bands were obtained using ChemiDoc MP System (BioRad, Benicia, CA, USA) a multiplex fluorescent and chemiluminescence system. The densitometric analyses were performed using ImageJ software (Wayne Rasband, National Institutes of Health, Bethesda, MD), and the values obtained represent the ratio between the immunodetected protein and Tubulin (the loading control). A detailed explanation on the quantification performed can be found in the supplementary file.

### Immunocytochemistry (ICC)

For ICC analysis, 1 × 10^4^ cells in 300ul DMEM/F12 were plated on coverslips placed on a 24-well plate in either normoxic or hypoxic conditions for 72 h. The cells were fixed using 4% PFA after achieving a desirable confluence. The cells were then rinsed with 1% PBS and permeabilization was done using 0.1% triton. The slips were then incubated in 5% BSA for 30 min to minimize nonspecific binding. Incubation with the primary antibody was done overnight at 4 °C with either anti-SOX2/OCT4 (Cell signaling technology, 1:400), anti-HIF-1alpha (Millipore, 1:500), anti-Glut-1 (Millipore, 1:500) anti-VEGF (Abcam,1:500:) anti-Survivin (Abcam, 1:500) anti-BCL2 (Abcam,1:500), anti-Ki67 (Dako, 1:150), anti-GFAP (DAKO, 1:500), and anti-Vimentin (Dako, 1:100). The slips were then rinsed using PBS before incubating with secondary antibodies conjugated with Alexa Fluor 488 (goat anti-mouse/rabbit; 1:2000) or Alexa Fluor 546 (goat anti-rabbit; 1:2000) for 2 h at room temperature. They were then washed with 1X PBS, before staining with DAPI. Finally, the slips were rinsed with PBS before mounting on glass slides using Fluoromount-G anti-fading agent (Emsdiasum). The cells were imaged using DMI8 advanced fluorescence microscopy (Leica Microsystems, Germany) and the fluorescence intensity of individual cells was measured and analysed using ImageJ software (NIH, USA). Negative controls were included in every ICC staining, where a selected number of slips (under both conditions) were stained with only the secondary antibody conjugated to the fluorophore that was used to adjust the microscope intensity, or baseline settings, before reading the results. A detailed explanation on the method employed for the ICC quantification is given in the supplementary file of this manuscript.

### RT-qPCR

Total RNA was extracted from GBM cells using Trizol Reagent (Ambion, Life Technologies) and the PureLink® RNA Mini Kit (Invitrogen, Thermo Scientific) following the manufacturer’s instructions. Sample RNA purity was estimated using (Nanodrop lite, Thermo Scientific) spectrophotometer where a ratio of 1.8 to 2 was considered pure. One microgram of the total RNA, pool RT microRNA primer (custom made) and SuperScript™ III Reverse Transcriptase (SS III, Invitrogen, Life technologies) were used to perform the cDNA synthesis. Quantitative real-time PCR (qRT-PCR) was done using Power SYBR green PCR master mix (Applied biosystems, Thermofisher scientific) and custom-made microRNA primers from Integrated DNA Technologies (IDT). The primers used are described in Table 1.

**Table 1:** Primers and sequences

Name	Forward primer (5′- 3′)	Reverse Primer (5′- 3′)
miR- 34-5p	ACACTCCAGCTGGGTGGCAGTGTCTTAGCT	CTCAACTGGTGTCGTGGAGTCGGCAATTCAGTTGAGAGACAACC
miR-128-3p	ACACTCCAGCTGGGTCACAGTGAACCGGTC	CTCAACTGGTGTCGTGGAGTCGGCAATTCAGTTGAGAGAAAGAGAC
miR-221-3p	ACACTCCAGCTGGGAGCTACATTGTCTGCT	CTCAACTGGTGTCGTGGAGTCGGCAATTCAGTTGAGAGGAAACCCA
miR- 17-5p	ACACTCCAGCTGGGCAAAGTGCTTACAGTG	CTCAACTGGTGTCGTGGAGTCGGCAATTCAGTTGAGAGCTACCTGC
miR-181a-3p	ACACTCCAGCTGGGACCATCGACCGUUGAT	CTCAACTGGTGTCGTGGAGTCGGCAATTCAGTTGAGAGGGTACAAT
miR-181b-5p	ACACTCCAGCTGGG- AACATTCATTGCTGTC	CTCAACTGGTGTCGTGGAGTCGGCAATTCAGTTGAGAGACCCACCG
miR-181c-5p	ACACTCCAGCTGGGAACATTCAACCTGTCG	CTCAACTGGTGTCGTGGAGTCGGCAATTCAGTTGAGAGACTCACCG
Universal reverse		CTCAAGTGTCGTGGAGTCGGCAA
SNU6	GCTTCGGCAGCACATATACTAAAAT	CTCAACTGGTGTCGTGGAGTCGGCAATTCAGTTGAGAGCGTTCCA
Human Actin	ATGAAGATCAAG ATCATTGCTCCT	ACATCTGCTGGAAGGTGGACA

The run was done using CFX96 Real-Time System (Bio-Rad). Melt curves were included in every run and the assays were performed in triplicates. Endogenous RNA U6 (RNU6) was used as a control for the normalization of mature miRNAs while actin was used for the for the normalization of the gene expression. Analysis of the miRNAs expression was calculated using the 2-^ΔΔ^Cq method [62]. The data was obtained from three independent experiments and analysed using Graph pad prism.

### Statistical analysis

The experimental data are expressed as the mean ± standard deviation (SD) of three independent replicates. Statistical differences between two groups were evaluated by the Student’s t-test. Those between more than two groups were subjected to analysis of variance (ANOVA) with Dunnett’s as a post-test using GraphPad Prism 5.0 statistics software (GraphPad Software, Inc., La Jolla, CA). *P* < 0.05 was considered statistically significant.

## Results

### The expression profile of miRNAs in 6 GBM cell lines compared to human astrocyte

**Fig. 1:**
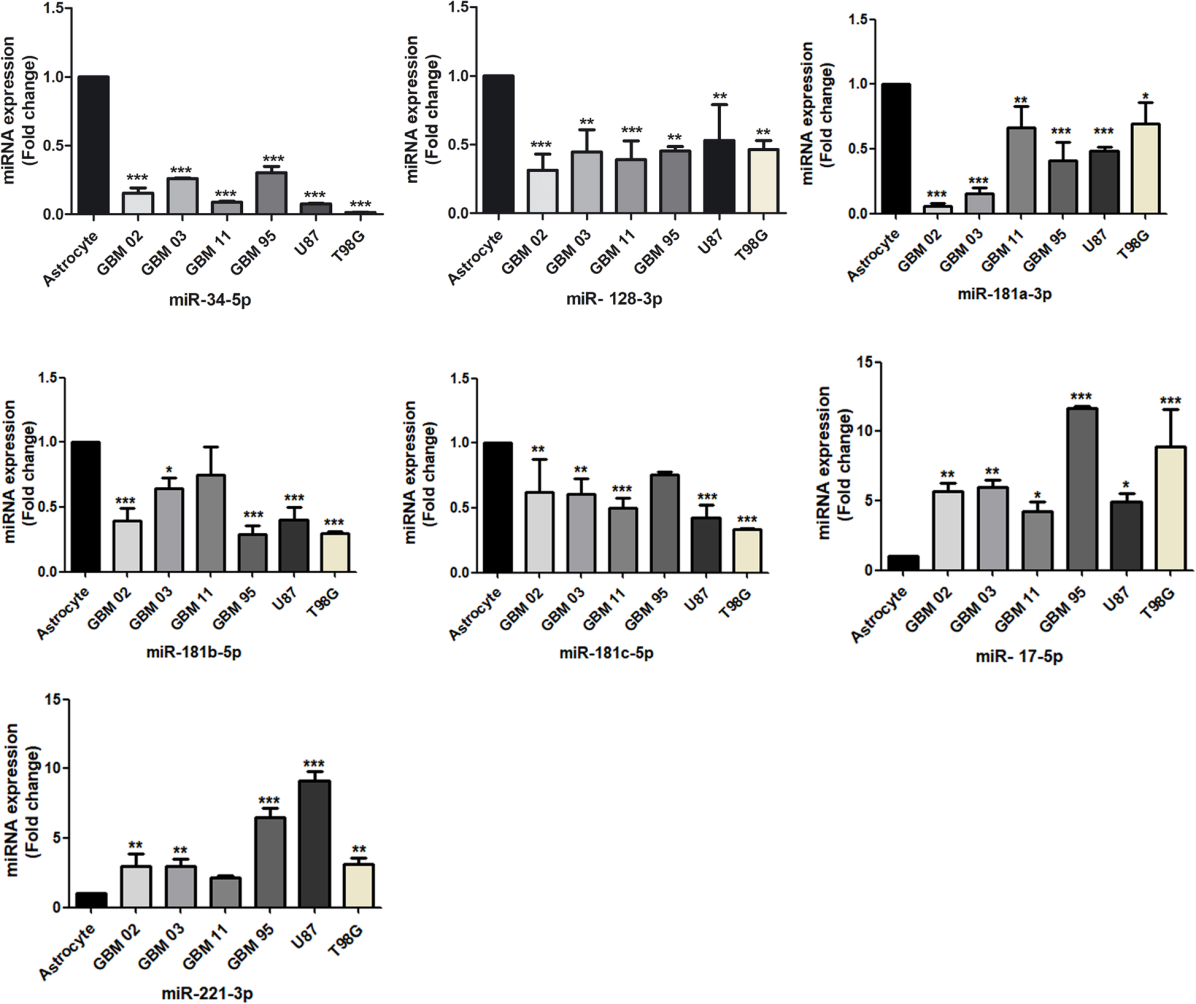
Expression of miRNAs in GBMs by qRT-PCR. The GBM cell lines were cultured under normoxic (21% O_2_) conditions. qRT-PCR was run using the SYBR green platform. The relative fold change difference was calculated using 2-^ΔΔ^CT method where RNU6 was used as the normalizer. The data was analysed using 1-way ANOVA with Dunnett’s as a post-test and drawn using Graph pad prism. Each value represents the mean ± SD of three independent runs and * indicates *p* < 0.05; compared to the astrocyte’s cells

Dysregulated expression of miRNAs has been observed in GBM making them a promising diagnostic biomarker [20]. Similarly, miRNAs expression is tissue or cell-specific and this expression profile has not been established for our GBM cell lines. We evaluated seven miRNAs reported from previous studies, miR-34 [21, 22], miR-128 [29–32], miR-181 family [30, 31, 37], miR-221 [30, 31] and miR-17 [49], as either upregulated or downregulated in glioblastoma samples. Therefore, we cultured the GBM cell lines and a human astrocyte control cell line under normoxic conditions for 72 h and performed the qRT-PCR using the SYBR green platform. Results from our study found miRNAs 34-5p, 128-3p, 181a/b/c to be downregulated while miRNAs 221- 3p and 17-5p to be upregulated when compared to the astrocytes. Among all the cell lines analysed, the miRNA expression profile was more homogenous for miR-34-5p and 128-3p **(**Fig. 1**).** Our results agreed with previous reports.

### The effect of hypoxia on the expression of markers of GBM aggressiveness

Hypoxia is a GBM common phenomenon and to cope with low oxygen availability, intracellular modifications occur in the cells primarily via the HIF transcription factor [16]. For our study, a hypoxia culture model was used to create a hypoxic niche in order to reflect what happens in the brain microenvironment of a patient with GBM [57]. Thus, we used this model to understand the cellular mechanisms of the GBM cells in vitro. The cells were cultured under normoxia (21% oxygen) and under hypoxia (1% oxygen) for 72 h, and different factors associated with GBM aggressiveness were investigated as follows.

#### Cells acquired hypoxia as confirmed by HIF-1α translocation to the nucleus

**Fig. 2:**
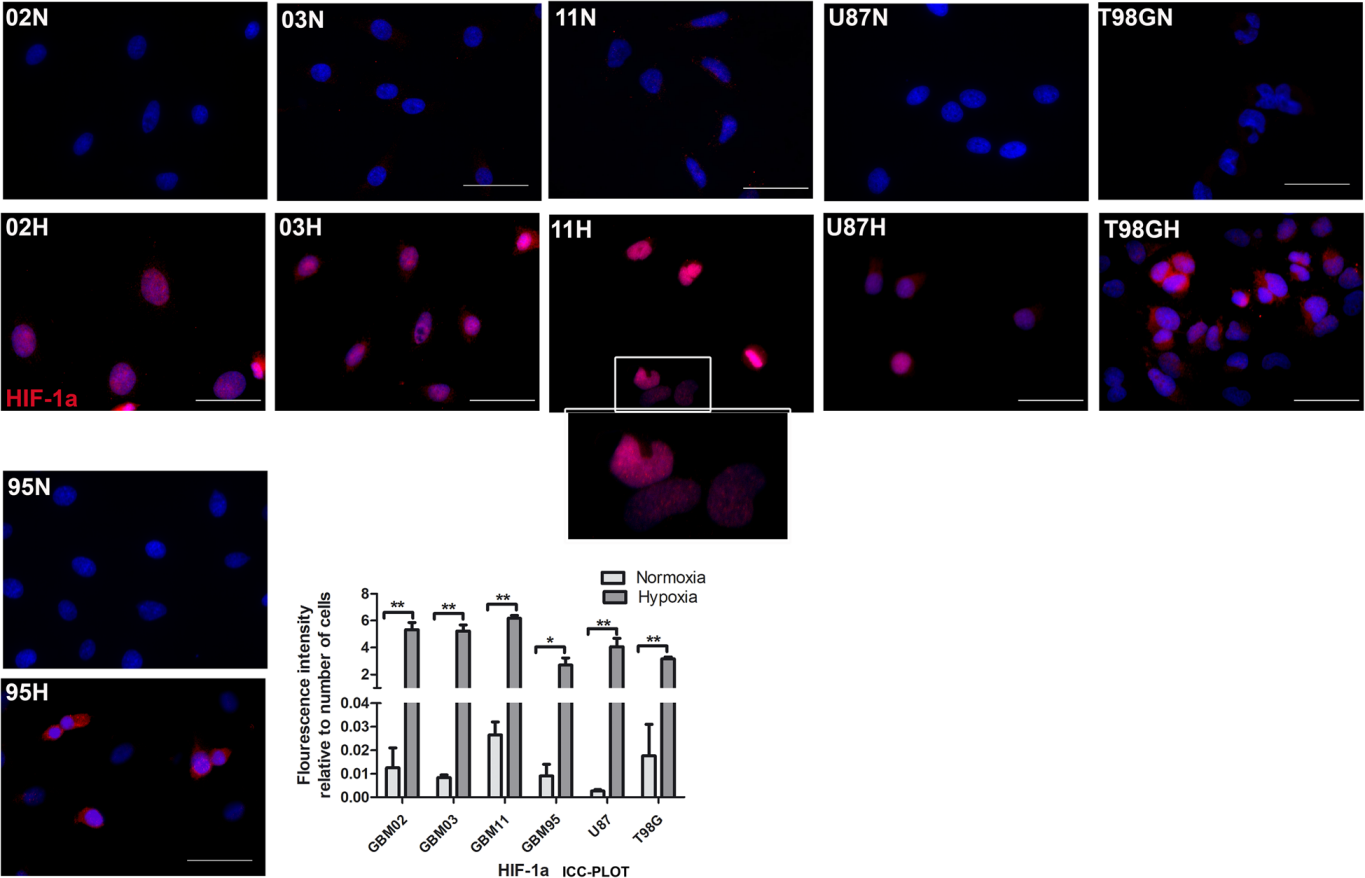
Translocation of HIF-1α into the nucleus after the hypoxia acquisition. GBM cell lines were cultured under normoxia (N) and another set-in hypoxia (H) for 72 h and stained using HIF-1α by immunocytochemistry. The Images were taken with DMi8 Leica microscope and prepared and quantified using ImageJ graph pad prism. Analysis was done using student t test to compare the expression of HIF-1α between each cell line under normoxia verses hypoxia. Each value represents the mean ± SD of three independent experiments, * indicates *p* < 0.05. Scale bar = 50 μm

Before evaluating the effects of hypoxia, we had to confirm the acquisition of hypoxia by the cells. It is known that under hypoxia, the HIF alpha subunit is usually stabilized and translocated from the cytoplasm to the nucleus where it heterodimerizes with the beta subunit [16, 17]. Based on this fact, our cells showed a significant HIF-1α translocation in the nucleus by immunocytochemistry staining confirming that our cells acquired the hypoxia microenvironment **(**Fig. 2**).**

#### Hypoxia microenvironment induced morphological changes in GBM cells

**Fig. 3:**
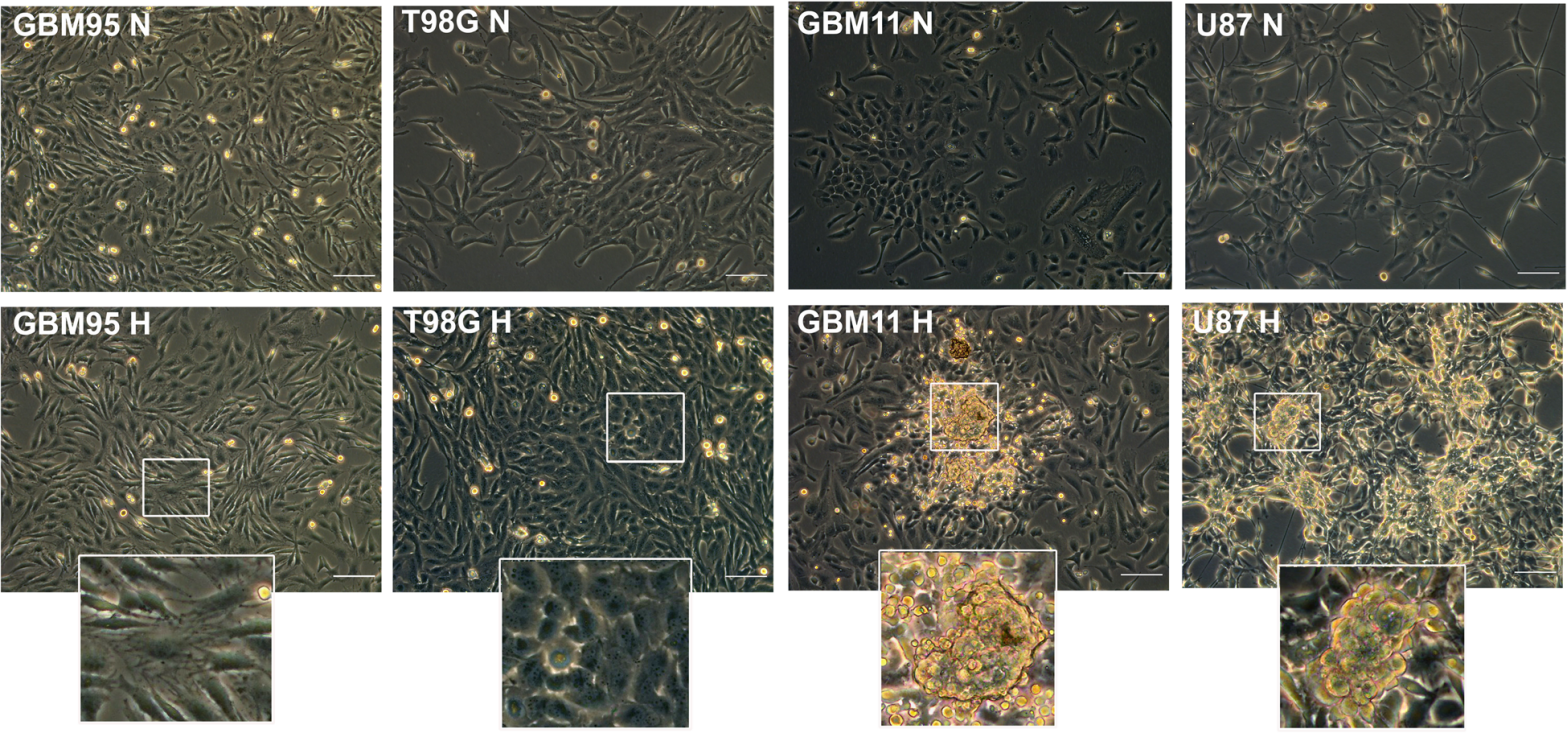
Morphological changes of cells under hypoxia microenvironment. GBM cells (GBM02, GBM03, GBM11, GBM95, U87 and T98G) were cultured in DMEM/F12 with 10% FBS. One batch of the cells was maintained at 37 °C in a humidified 5% CO2 and 95% air atmosphere for normoxia (N) while the other at 1% O2 for hypoxia (H). The photos were taken using DMI 8 Leica microscope and edited using image J. Scale bar = 200 μm

Cells have been reported to undergo intracellular modifications to cope with low oxygen availability. To test this fact, we cultured cells under normoxia (21% O_2_) and under hypoxia (1% O_2_) and photographed them after 72 h of cell culture without interference. Our results suggested the cells to be viable under both micro-environmental conditions with notable changes in GBM11, GBM95, U87 and T98G under hypoxia (H) induction. The GBM95H had a population of cells with fibrous-like edges. The T98GH had cells with a reduced spindle morphology or elongations as compared to their counterparts grown in normoxia. The GBM11H and U87H had an outstanding phenotypic morphology consisting of clustered or sphere-like cells with “well-defined borders” resembling to what we observe when we culture the GBM cells in stem-cell defined media during the initial days of transformation into an undifferentiated state. There were no notable morphological differences in GBM02 and GBM03 (data not shown) **(**Fig. 3**).**

#### Hypoxia induced increased expression of stem cell markers

**Fig. 4:**
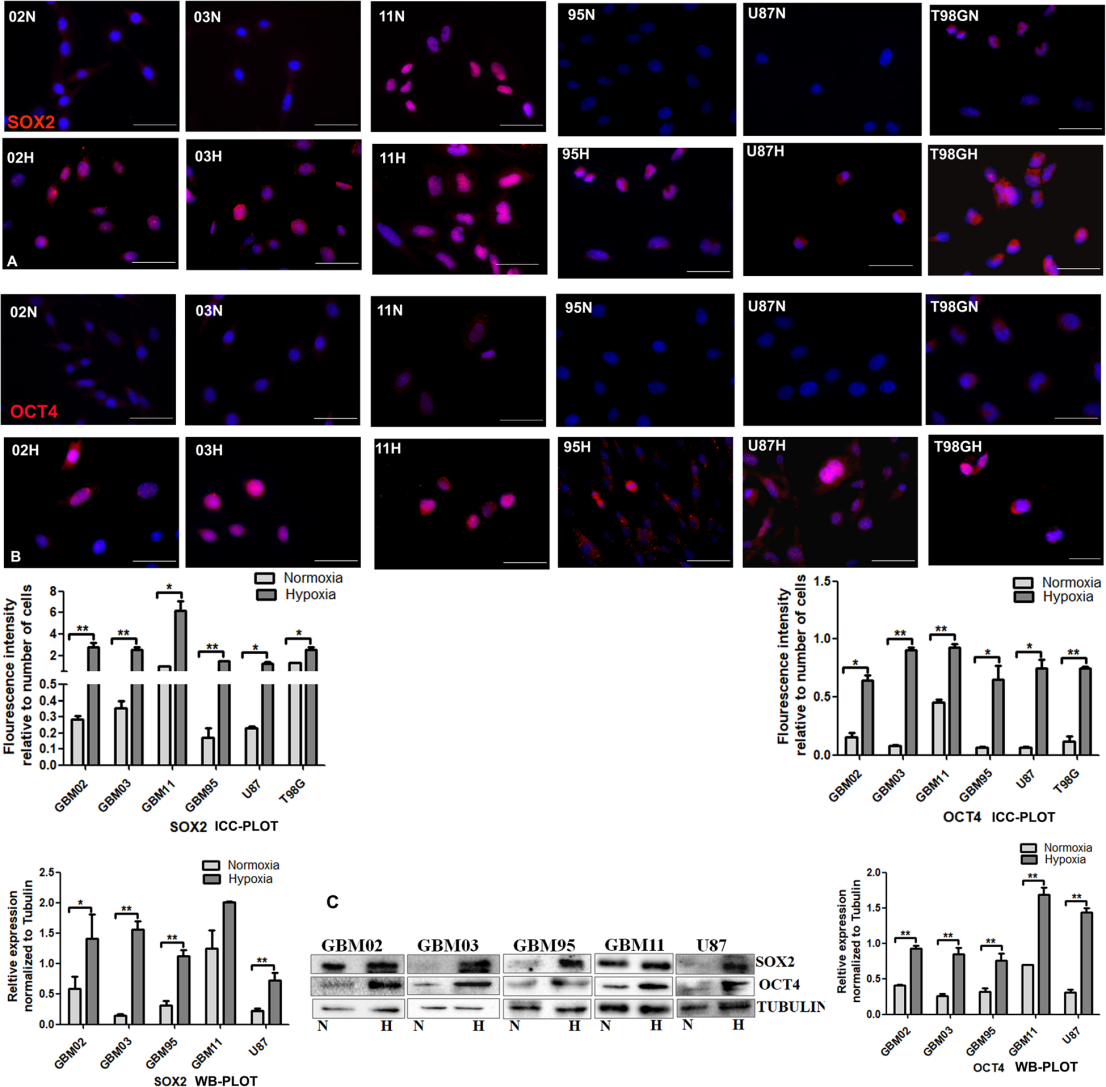
Enhanced the expression of the stem cell markers. **A** & **B.** Cells were cultured under normoxia (N) and in hypoxia (H) for 72 h and stained for Sox2 and Oct4 by immunocytochemistry. The Images were taken with DMi8 Leica microscope and prepared and quantified using ImageJ. **C**. Western blotting was done by the SDS-PAGE method and densitometry done using ImageJ. The relative expression was normalized to tubulin. Analysis was done using student t test using graph pad prism. Each value represents the mean ± SD of three independent experiments, * indicates *p* < 0.05. Scale bar = 50 μm. Representative Western blots are shown as cropped images. Full-length blots are presented in Additional File 2 (Fig. S2)

Hypoxia has been associated with reprogramming towards a stem-like cell phenotype and an increased expression of the stem-cell markers [15]. To study this effect, we cultured our cells under hypoxia microenvironment and analysed the differential expression of the stem cell markers, SOX2 and OCT4. Our results showed an upregulated expression of SOX2 and OCT4 in all the cells under hypoxia conditions (Fig. 4a**- c**). The high expression of SOX2 and OCT4 was also observable in GBM11N by immunocytochemistry (Fig. 4a & b).

#### Hypoxia increased GLUT-1 associated with metabolism

**Fig. 5:**
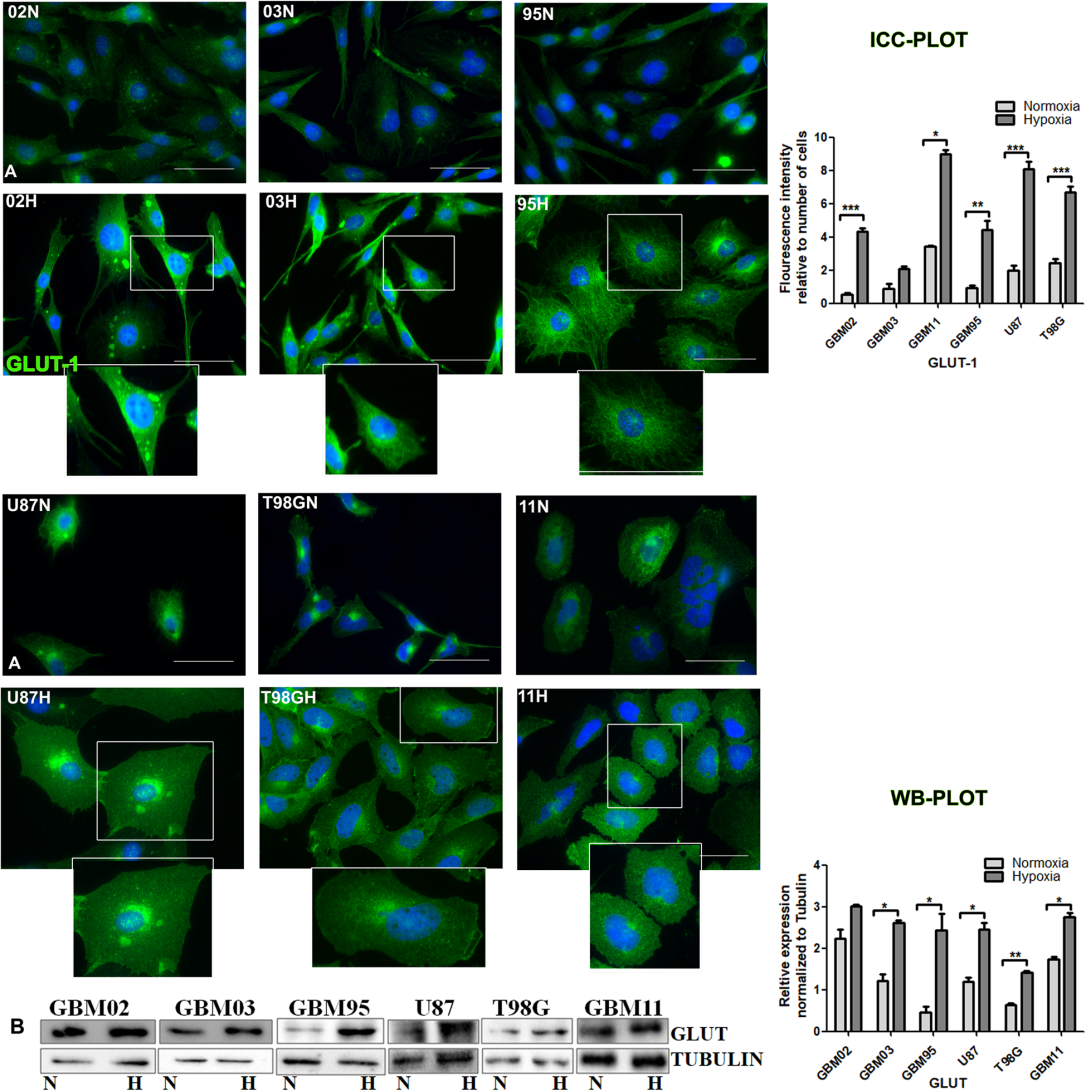
Enhanced expression of GLUT-1 under hypoxia microenvironment. **A.** Cells were cultured under normoxia (N) and another set in hypoxia (H) for 72 h and stained for GLUT-1 by immunocytochemistry. The Images were taken with DMi8 Leica microscope and quantified using ImageJ. **B.** Western blotting was done by the SDS-PAGE method and densitometry done using ImageJ. The relative expression was normalized to tubulin. Data was analysed using student t test by graph pad prism. Each value represents the mean ± SD of three independent experiments, * indicates p < 0.05. Scale bar = 50 μm. Representative Western blots are shown as cropped images. Full-length blots are presented in Additional File 3 (Fig. S3)

The glucose transporter 1 (GLUT-1), plays a role in metabolism during glycolysis by facilitating the entry of glucose into the cytoplasm of the cell. In the context of limited oxygen, the cell makes up for the limited energy by increasing its glucose supply that would translate to increased GLUT-1. To test this fact, we evaluated the expression of GLUT-1 in our cells cultured under both the microenvironments. Data from our work showed an expression of GLUT-1 under both microenvironments. Hypoxia induced an upregulation of GLUT-1 in all our tested cells **(**Fig. 5a & b**).** We also observed different staining patterns between our cells, where GBM 02H, 03H and 95H showed a different staining pattern from the one observed in U87H, T98G and GBM11H. The U87H, T98G and GBM11H had suggestively larger cells **(**Fig. 5a**).**

#### Hypoxia increased VEGF associated with angiogenesis

**Fig. 6:**
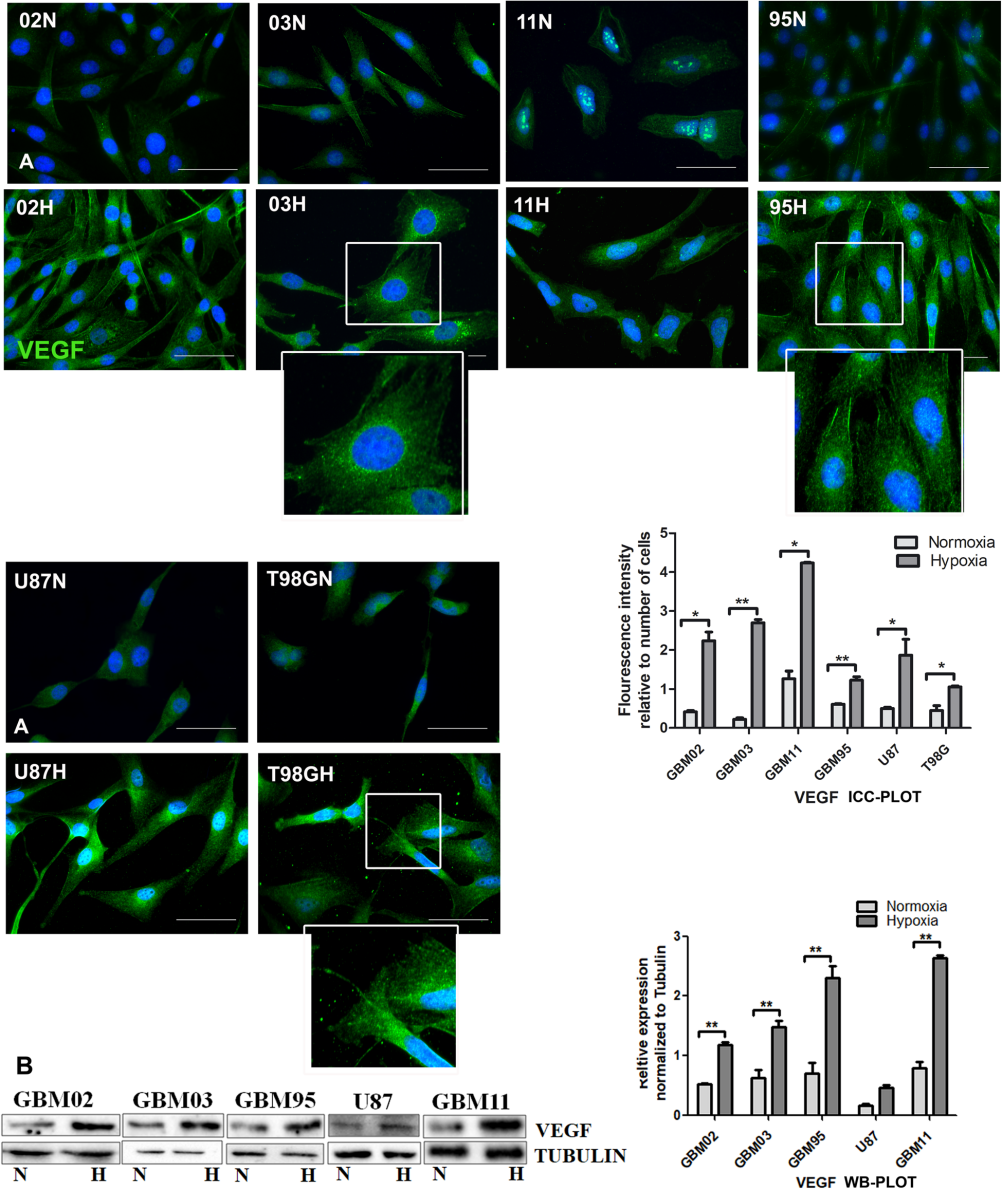
Enhanced expression of VEGF under hypoxia. **A**. Cells were cultured under normoxia (N) and another set in hypoxia (H) for 72 h and stained for VEGF by immunocytochemistry. The Images were taken with DMi8 Leica microscope and quantified using ImageJ. **B**. Western blotting was done by the SDS-PAGE method and densitometry done using ImageJ. The relative expression was normalized to tubulin. The graphs were drawn and analysed using t test by graph pad prism. Each value represents the mean ± SD of three independent experiments, * indicates p < 0.05. Scale bar = 50 μm. Representative Western blots are shown as cropped images. Full-length blots are presented in Additional File 4 (Fig. S4)

Altered microvasculature is a hallmark of GBM. GBMs proliferate, and expand beyond their existing blood supply, creating hypoxic niches. This results in the upregulation of angiogenic factors like VEGF that induces the formation of blood vessels. We analysed the differential expression of VEGF, a pro-angiogenic factor, in both conditions. All our GBM cells expressed VEGF as reported but the expression was further upregulated under the hypoxia microenvironment **(**Fig. 6a & b**).**

#### Increased expression of the anti-apoptotic and proliferation markers under hypoxia

**Fig. 7:**
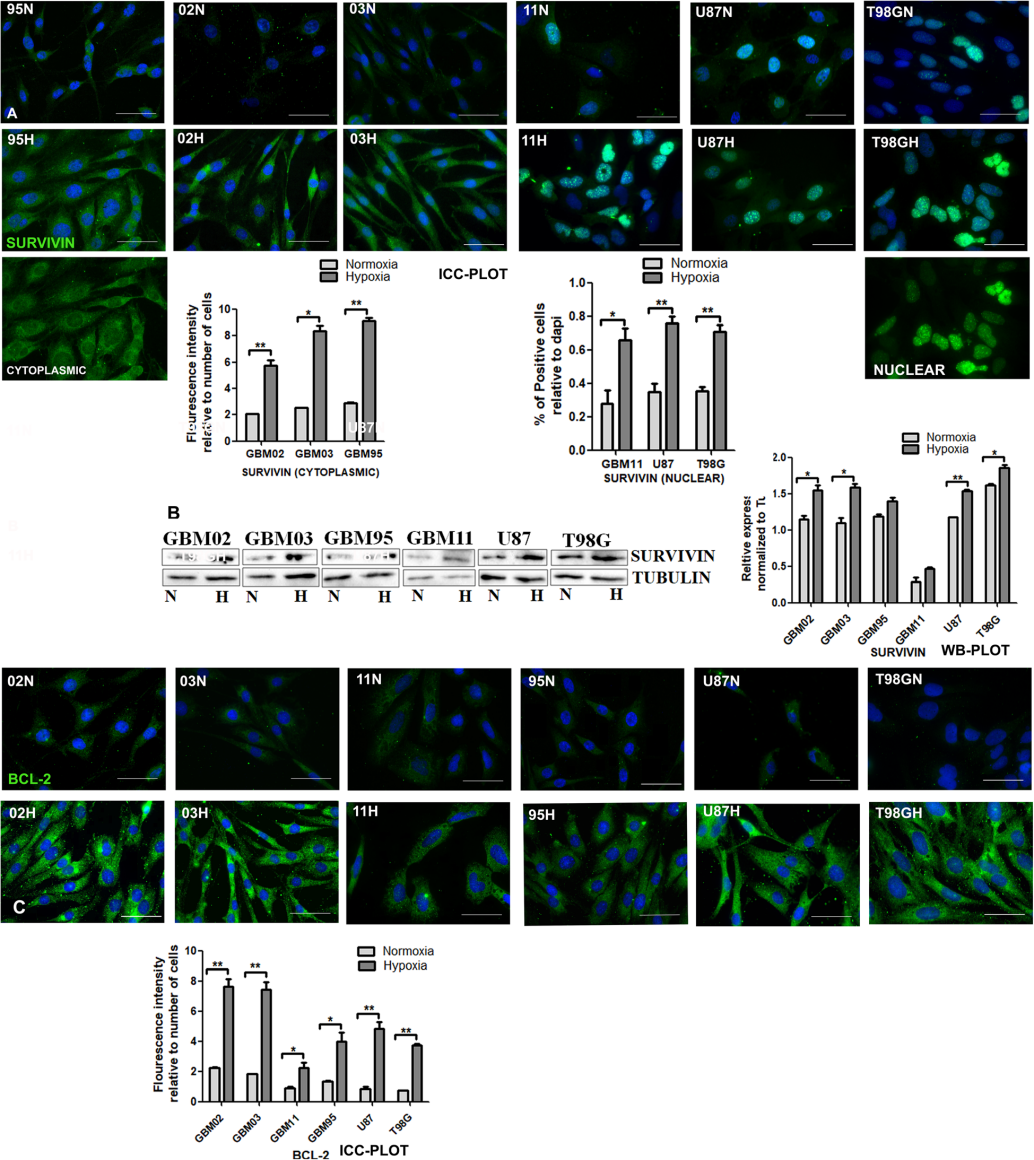
Increased anti-apoptotic and proliferation markers under hypoxia. **A** & **C.** Cells were cultured under normoxia (N) and another set in hypoxia (H) for 72 h and stained for Survivin and Bcl2 by immunocytochemistry. The Images were taken with DMi8 leica microscope and prepared and quantified using ImageJ. The relative expression was normalized to tubulin. The data was analyzed using student t test by graph pad prism. **B**. Western blotting was done by the SDS-PAGE method and densitometry done using ImageJ. The relative expression was normalized to tubulin. The graphs were drawn and analyzed using t test by graph pad prism. Each value represents the mean ± SD of three independent experiments, * indicates p < 0.05. Scale bar = 50 μm. Representative Western blots are shown as cropped images. Full-length blots are presented in Additional File 5 (Fig. S5)

GBM’s aggressiveness is partially attributed to its decreased apoptosis or apoptotic escape. We evaluated the expression of two anti-apoptotic proteins survivin and Bcl-2 to test this hypothesis. All cells expressed the anti-apoptotic factors in both microenvironments, but the expression was enhanced further under hypoxia **(**Fig. 7a-c**).** Survivin is also a member of the inhibitor of apoptosis protein (IAP) family and is expressed either as a cytoplasmic or nuclear pool, being involved in the regulation of cell division and apoptosis [63]. We were able to observe the differential expression of survivin in our cells. GBM02, 03 and 95 expressed survivin as a cytoplasmic pool while GBM11, U87 and T98G expressed survivin as a nuclear pool **(**Fig. 7a**).** Cytoplasmic survivin has been shown to have antiapoptotic properties [64] while nuclear survivin has been highlighted to play an important role in chromosomal segregation during mitosis spindle [65]. The Bcl-2 expression was localized in the cytoplasm and was found upregulated in our cells under hypoxia (Fig. 7c). Ki-67 is a nuclear protein that is preferentially expressed during all active phases of the cell cycle (G1, S, G2 and M-phases) but is absent in resting cells (Go-phase). We evaluated the expression of Ki-67, a marker of proliferation, from our cell lines and the expression was clearly visible in all our cells under normoxia and hypoxia suggesting that our cells did not go into the resting or quiescent phase during the 72 h of culture (Sup Fig. 1).

#### Enhanced expression of cytoskeleton proteins

**Fig. 8:**
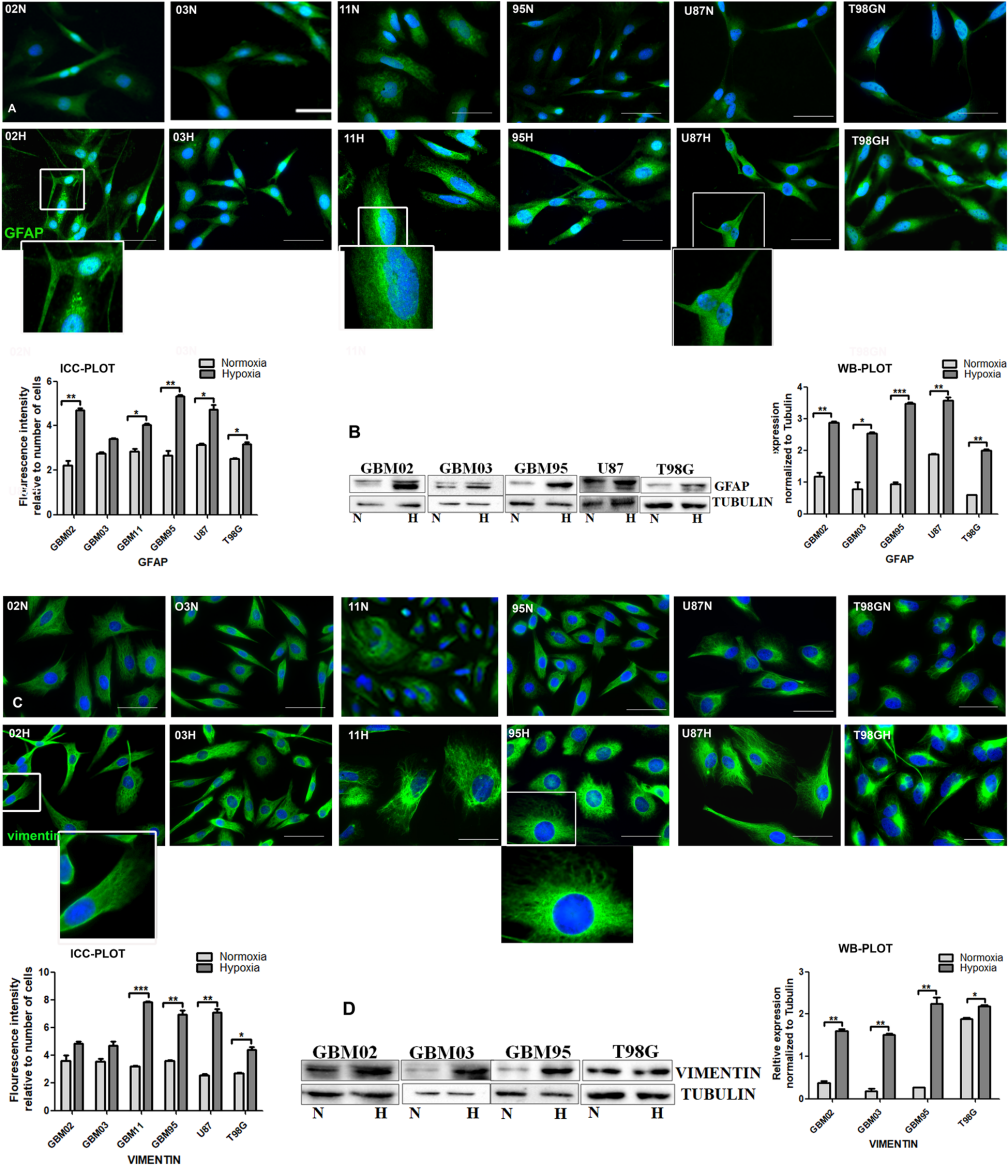
Increased expression of an astrocytic and a cytoskeleton protein hypoxia. **A** & **C.** Cells were cultured under normoxia (N) and another set in hypoxia (H) for 72 h and stained for GFAP) and vimentin by immunocytochemistry. The Images were taken with DMi8 Leica microscope and prepared and quantified using ImageJ. **B** & **D.** Western blotting was done by the SDS-PAGE method and densitometry done using ImageJ. The relative expression was normalized to tubulin. The graphs were drawn and analysed using t test by graph pad prism. Each value represents the mean ± SD of three independent experiments, * indicates p < 0.05. Scale bar = 50 μm. Representative Western blots are shown as cropped images. Full-length blots are presented in Additional Files 6 & 7 (Fig. S6 and S7)

Glial fibrillary acidic protein (GFAP) is a class III intermediate filament (IF) protein that constitutes a portion of the cytoskeleton. It is a cell-specific marker that, during the development of the central nervous system, distinguishes astrocytes from other glial cells. All our GBM cell lines expressed GFAP as expected since they are of astrocytic origin **(**Fig. 8a & b**)**. On the other hand, vimentin is an intermediate filament protein type III, which also forms part of the cytoskeleton of vertebrate cells and is expressed by proliferating cells. Our results also showed vimentin to be significantly expressed in all GBM cell lines **(**Fig. 8c and d**).** Both GFAP and vimentin were suggestively upregulated under the hypoxic microenvironment.

### Hypoxia microenvironment induced dysregulation of miRNAs expression

**Fig. 9:**
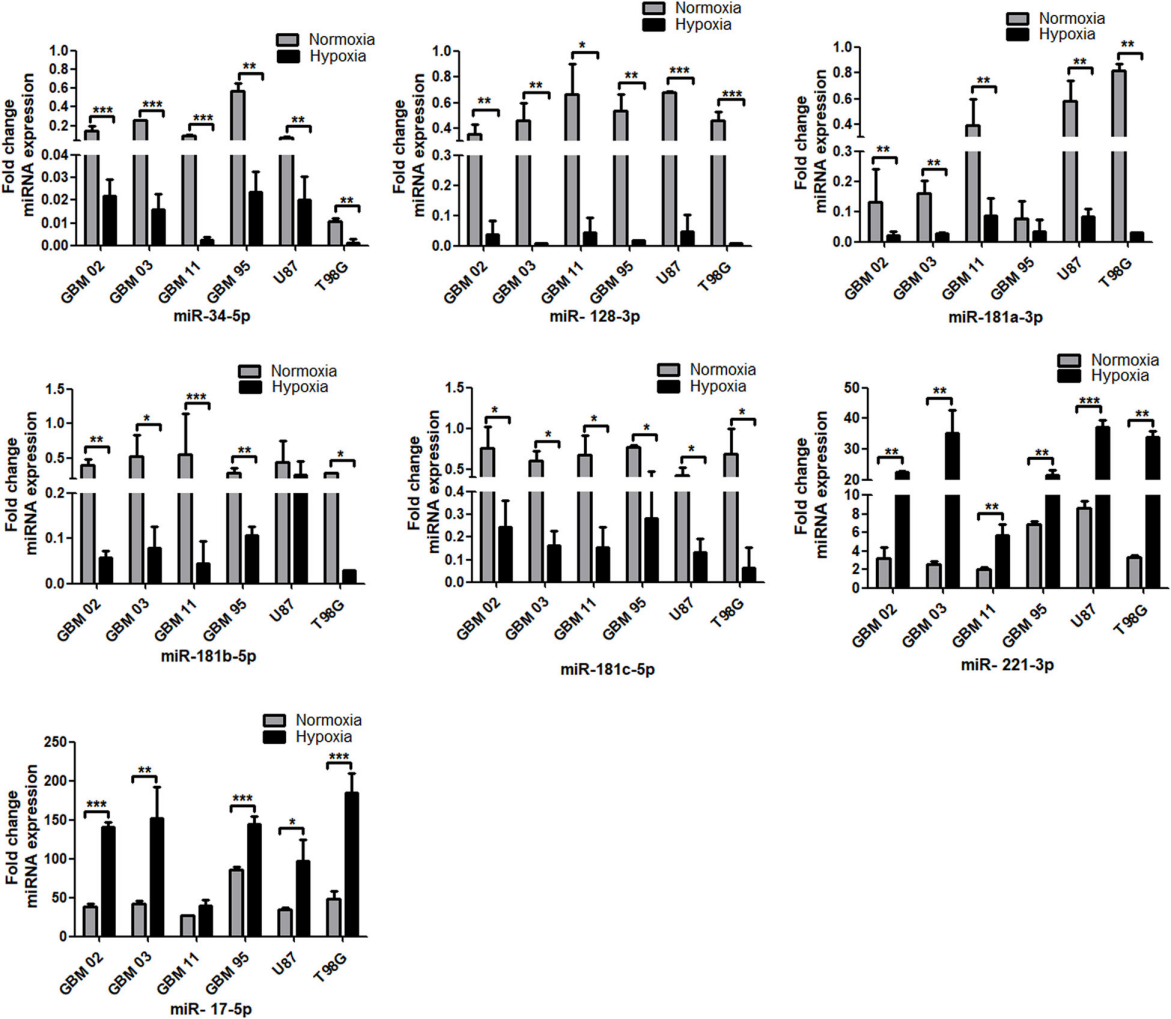
Dysregulation of miRNAs in GBMs under hypoxia by RT-qPCR. The GBM cell lines were cultured under hypoxic conditions. RT-qPCR was run using the SYBR green platform. The relative fold change difference was calculated using 2-^ΔΔ^Cq method where RNU6 was used as the normalizer. The graphs were drawn and analysed using t test by graph pad prism. Each value represents the mean ± SD of three independent runs and * indicates p < 0.05; compared to the astrocytes grown in either normoxia or hypoxia conditions

Hypoxia microenvironment has been documented to alter the expression of miRNAs [56]. To investigate this hypothesis, cell lines were cultured again under normoxia (21% O_2)_ and hypoxia (1% O_2_) for 72 h. Results in Fig.1 show miR-34-5p, 128-3p and 181a/b/c to be downregulated while miR-221-3p and 17-5p to be upregulated when a comparison was made between a GBM and a non GBM cell line (astrocytes). After hypoxia induction, miR-34-5p and miR-181a/b/c had a further 2–3-fold downregulation while miR-128-3p had a 4–5-fold downregulation. Similarly, miR-17-5p and 221-2p were further upregulated by a 3-fold **(**Fig. 9**)**.

## Discussion

Glioblastoma is a fatal type of primary adult brain tumour with a dismal overall prognosis [1, 2]. The molecular mechanisms underlying the GBM aggressiveness are still unclear. In this study, we investigated the expression profile of specific miRNAs, stemness and survival genes in GBMs grown under normoxia or hypoxia conditions. Results from our study show miR-34-5p, 128-3p and 181a/b/c to be downregulated whereas miR- 221- 3p and 17-5p to be upregulated when compared to astrocytic cells, a non-GBM control. These results agree with what has been reported previously.

Hypoxia is a main feature of GBM [10, 11] being associated with poor prognosis [9]. Many intracellular modifications allowing cell adaptation to the low O_2_ availability are primarily controlled by the transcription factor system of the hypoxia-inducible factors (HIFs) [66–68]. In agreement with other studies, we were able to observe an increased expression of HIF-1α when we cultured our cells under hypoxia microenvironment, consequently resulting to an increased expression of SOX2, OCT4, VEGF, GLUT-1, Bcl-2, survivin, GFAP and vimentin. The upregulation of HIF under hypoxia microenvironment has been shown to induce the expression of known pluripotent stemness factors, such as KLF4, MYC, OCT4, SOX2, and NANOG [69], to reprogram towards a cancer stem cell (CSC) phenotype and to influence the expansion of CSC populations [70]. This is similar to our observations where we found an increase in stemness factors, OCT4 and SOX2, which were upregulated under hypoxia. Additionally, hypoxia microenvironment has been shown to induce metabolic reprogramming [71, 72], alter angiogenesis [73] and induce cells apoptotic escape [74, 75]. Findings from our study showed an increased expression of GLUT-1 that plays a role in metabolism, increased VEGF that plays a role in angiogenesis, increased BCL-2 and survivin that plays a role in apoptotic escape, all of which were further upregulated under hypoxia microenvironment. Additionally, our results showed differential expression of survivin either as a cytoplasmic or a nuclear pool. The significance, or potential functions of surviving, in different cellular localizations is still a matter of debate. While cytoplasmic survivin has been shown to have anti-apoptotic properties [76], nuclear survivin has been shown to play a role in mitosis [77]. An inverse correlation between the cytoplasmic expression of survivin and cell apoptosis in GBMs has been reported, thus supporting an anti-apoptotic function of the cytoplasmic form of survivin [78]. Shirai et al. reported nuclear survivin expression to be a poor prognosis predictor in GBM [79], while Saito and colleagues reported that the simultaneous expression of survivin in the nucleus and in the cytoplasm correlated with a poor prognosis in high-grade astrocytoma, including GBM [80]. More experiments would be needed to confirm the specific roles played by the two survivin expression pools in our cell lines.

It is important to highlight that HIF-1α can also be subjected to negative regulation by tumour suppressors such as Von Hippel-Lindau (VHL) and (PTEN) [51]. Additionally, miRNAs can also regulate the tumour suppressors that negatively regulate HIF-1α. This has been shown by a study that found that MiR-17 induced HIF-1α activation in response to stress by targeting PTEN in GBM cells. Inactivation of PTEN influenced the over-expression of HIF-1α, leading to cascade reactions in angiogenesis and migration [51]. Although we found an upregulated expression of miR-17 and HIF-1α, a correlation study between the two was not performed. Moreover, the PTEN expression profile has not been established from our cells to accurately link PTEN and HIF-1α. It is known from previous studies that the PTEN’s loss of function also results in HIF-1α activation by dysregulation of the PI3K/AKT pathway, especially in GBM cells [81]. Our results revealed an upregulation of HIF- 1α and miR-17 after hypoxia induction but a direct correlation between miR-17, PTEN and HIF-1α from our cell lines await further clarification.

We also show that hypoxia influenced the morphology of our GBM cells. There were observable morphological changes in GBM11, GBM95, U87 and T98G under hypoxia microenvironment. The GBM95H had a mixed population of cells with some showing fibrous-like edges, whereas the T98GH had cells with a reduced spindle morphology or elongations and were more clustered as compared to their counterparts grown in normoxia. The GBM11H and U87H had an outstanding phenotypic morphology consisting of clustered or sphere-like cells with “well-defined borders” with no evidence of necrosis. The GBM11H and U87H clustered morphology was like what we observed when culturing GBM cells in stem cell defined media during the early days of conversion from a differentiated to an undifferentiated state. There are limited studies describing the accurate effects of hypoxia on the morphological aspects of the cells. A study by Li and colleagues showed that after incubation of U87 cells, U251 cells and 02GBM in 1% O_2_ for 72 h, the processes were absent, and oval cells aggregated in masses with no evidence of necrosis [82]. Similarly, a study that exposed U87, SNB75 and U251 cells to hypoxia (1% O_2_) for 72 h found a marked morphological difference in U87 and SNB75 where the cells had a more elongated morphology and were more loosely clustered than in normoxia cultured cells [83]. More studies on this area are needed to accurately explain the morphological changes observed under the hypoxia microenvironment.

**Fig. 10:**
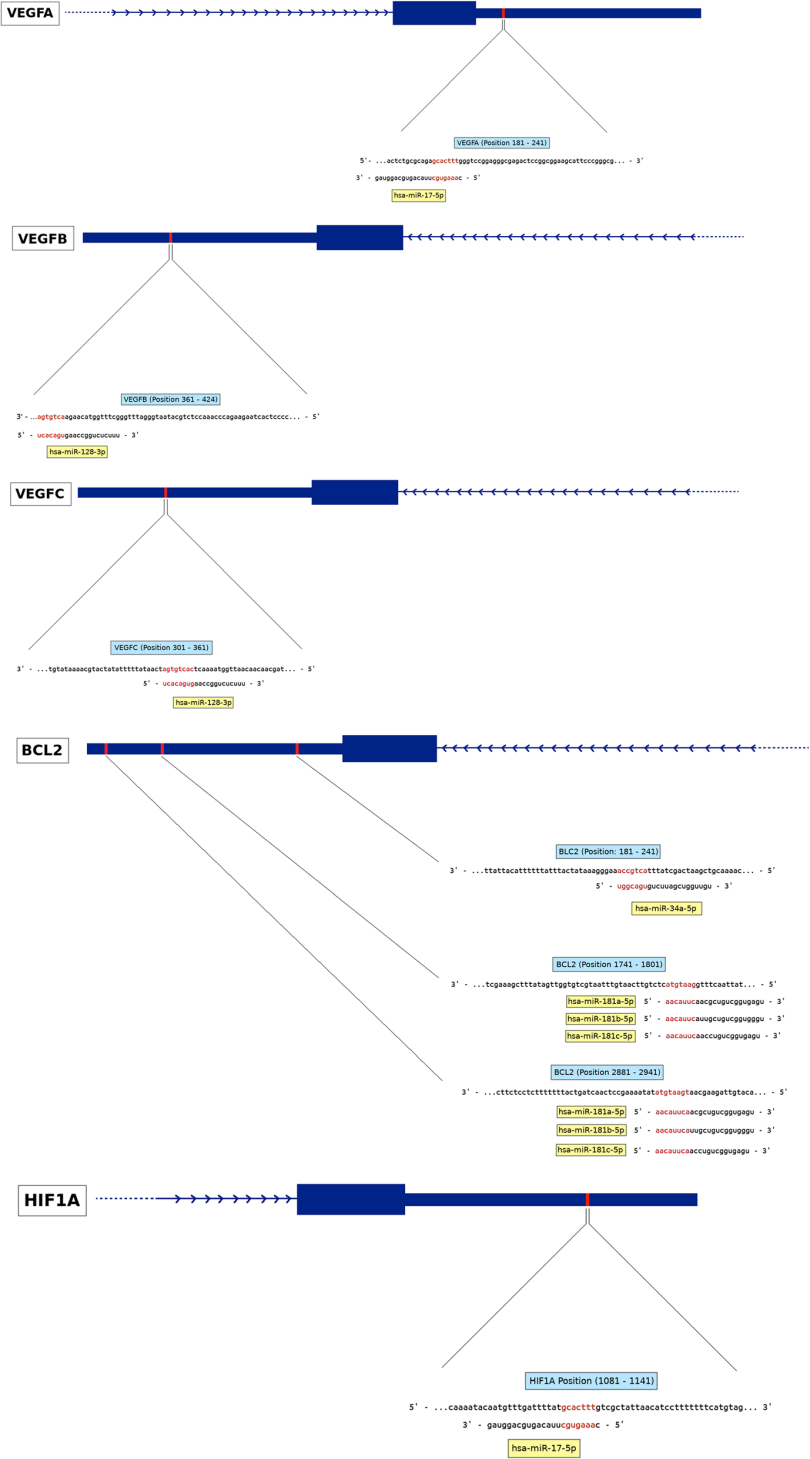
Bioinformatic analysis of the inter-relationship between the study miRNAs and genes. By using miRDB and TargetScan we confirmed previous findings showing that target sequences for miR-34-5p and miR-181a-5p, miR-181b-5p and miR-181c-5p are present in the 3′ UTR of the BCL-2 gene. Similarly the targets for miR-17-5p were found in the 3′ UTR of HIF1A and VEGF-A. The target sequences for miR-128-3p were present in the 3’UTR of VEGF-B and VEGF-C

Studies suggest that stress signals are responsible for altered miRNA expression and functions [84–86]. Some miRNAs can modify gene expression by cross-talking with the tumour micro-environment, and their expression can be altered in turn by distinct stress conditions such as hypoxia, oxidative stimulation or radiation [87–89]. Hypoxia inducible factor 1 has been hypothesized to regulate a panel of miRNAs, whereas some of miRNAs also target HIF-1 [90]. Our data revealed that when the GBM cells were cultured under hypoxia conditions the levels of miR-34-5p, 128a-3p and 181a/b/c increased, while miR-221-3p and 17-5p were downregulated. A direct relationship between miRNA expression levels and the genes SOX2, OCT4, VEGF, GLUT-1, Bcl-2, Survivin, GFAP, Ki-67 and vimentin was evaluated using bioinformatics tools **(**Fig. 10**).** In agreement with previous studies, we found target sequences for miR-34-5p and miR-181a-5p, miR-181b-5p and miR-181c-5p in the 3′ UTR of the Bcl-2 gene suggesting that these miRNAs might directly regulate Bcl-2 and be responsible for its upregulation in hypoxia conditions. However, a target sites for miR-17-5p is present in the 3′ UTR of HIF1A and VEGF-A, yet both this miRNA and its targets are upregulated in GBM cells under hypoxia regimens. Furthermore, there was no direct inter-relationship between other genes and miRNAs investigated in this study. It is possible that these miRNA-based regulatory mechanisms might involve additional factors and control levels. For instance, increased expression levels of miR-17 in glioblastoma cultured under stress conditions are known to cause downregulation of the transcription factor PTEN. In turn, reduced PTEN results in the upregulation of HIF1α and VEGF [51]. Therefore, additional experiments will be necessary to fully understand the relationship between the miRNAs and protein-coding genes that we find to be affected by hypoxia conditions in GBM cells. The tumour-suppressor protein, p53, lies at a nexus of cellular pathways that sense DNA damage, cellular stress (nutrient deprivation, hypoxia etc), oncogenic activation and improper mitogenic stimulation [91]. In response to such signals, p53 induces growth arrest, promotes apoptosis, blocks angiogenesis, or mediates DNA repair in a context-dependent manner [92]. In this sense, p53 has been shown to repress miR-17-92 transcription and consequently sensitizing cells for hypoxia-induced apoptosis [93]. However, our results have found miR-17 belonging to the miR-17-92 cluster and upregulated under hypoxia.

Genes encoding miRNAs in the miR-34 family are direct transcriptional targets of p53, whose induction by DNA damage and oncogenic stress depends on p53 expression both in vitro and in vivo. Studies have shown that ectopic expression of miR-34 induces cell cycle arrest in both primary and tumour-derived cell lines [27] which is consistent with the observed ability of the miR-34 family to play a role in the TP53 tumour suppressor function causing cell cycle arrest and apoptosis [24, 27, 94]. Similarly, p53 that acts as a negative transcriptional regulator of the miR-17-92 cluster and a positive transcriptional regulator of the miR-34 is found mutated in a variety of GBM [95, 96]. Interestingly, miR-128 has been reported to be downregulated in response to hypoxia [90] which is in agreement with our observation. Despite the emergence of numerous studies on hypoxia-regulated miRNAs (HRMs) the function of HRMs remains to be elucidated. The correlation between the tumour suppressors and the oncogenes with the miRNAs from our cell lines awaits further confirmation. Similarly, we aim to test patient samples in the future.

The evidence provided from the present study highlights the influence of the hypoxia microenvironment on various aspects of the GBM tumour cells and this may help to advocate for the laboratory experiments to be conducted in a microenvironment reflective of the tumour’s niche, thus representing a more accurate method.

**Fig. 11:**
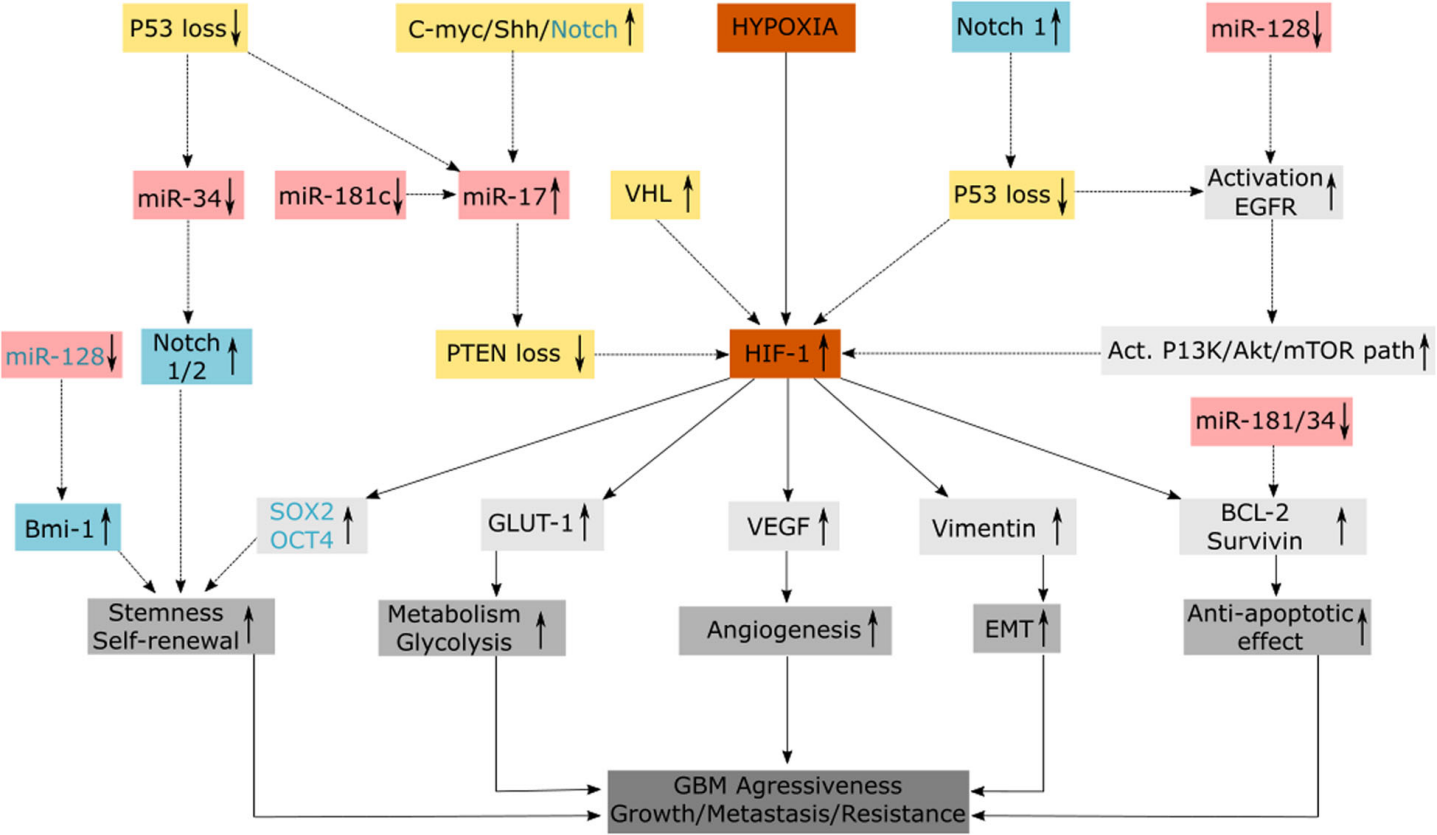
Summary figure. The continuous arrows show findings derived from our results while the dotted arrows represent the summary obtained from previous published works. Under hypoxia microenvironment, HIF-1α was upregulated similar to its downstream targets associated with stemness (SOX2, OCT4), metabolism (GLUT-1), angiogenesis (VEGF), EMT transitioning (Vimentin) and anti-apoptosis (Bcl-2, survivin). The miR-34, 128 and 181 were found downregulated from our study similar to what has been reported. Previous works have associated the same miRNAs with key tumour suppressor or oncogenes which can also influence their activation directly or indirectly. Additionally, the miRNAs can also influence the expression of the tumour suppressor or oncogenes. In this manner, miR-34 has been reported to target multiple oncogenes including c-MET, NOTCH 1 and 2, BCL-2 and CDK6 in gliomas. Additionally, P53 has been shown to influence miR-34 expression. An oncogene c-myc has also been reported to directly activate the transcription of miR-17 family. Oncogenic signalling pathways, such as those of Notch and Sonic Hedgehog, have also been shown to activate miR-17-92 in cancer. Most of the key tumour’s suppressors are known to have loss of function mutations that could also influence the expression of the miRNAs and the HIF. That means HIF-1α can also be subjected to the negative regulation by tumour suppressors such as Von Hippel-Lindau (VHL) and (PTEN). Additionally, some miRNAs can regulate the tumour suppressors that negatively regulate the HIF-1α. This has been shown by a study that found that MiR-17 induced HIF-1α activation in response to stress by targeting PTEN in GBM cells. Also, p53 that acts as negative transcriptional regulator of the miR-17-92 cluster under hypoxia is found mutated in the GBM

Figure 11 summarises all the results for a clear comprehensive overview.

## Conclusions

This study demonstrated a functional link between hypoxia, a well-documented tumour microenvironment factor, and miRNA expression. Moreover, we show that hypoxia controls the expression of several tumour genes that play a role in stemness, metabolism, angiogenesis, and anti-apoptosis, which are all involved in GBM tumorigenesis. Taken together, our findings comprehensively illustrate the effect of the hypoxia microenvironment on GBM cells in vitro, but in vivo studies are still required to compare the specific mechanism underlying this observation. Hypoxia culture could represent a closer culture model reflective of the brain microenvironment of a patient with glioblastoma, a factor that should be considered when conducting assays in the pursuit of identifying therapeutic targets and novel treatments.

## Supplementary Information

**Additional file 1 **Expression of the marker of proliferation under different microenvironments. **A.** Cells were cultured under normoxia (N) and another set in hypoxia (H) for 72 h and stained for KI67. The Images were taken with DMi8 Leica microscope and prepared and quantified using ImageJ. Western blotting was done by the SDS-PAGE method and densitometry done using ImageJ. The relative expression was normalized to tubulin. The graphs were drawn and analysed using t test by graph pad prism. Each value represents the mean ± SD of three independent experiments, * indicates *p* < 0.05. Scale bar = 50 μm.

**Additional file 2 **: **Fig. S2**. Full-length Western-blots for Fig. 4C.

**Additional file 3 **: **Fig. S3**. Full-length Western-blots for Fig. 5B.

**Additional file 4 **: **Fig. S4**. Full-length Western-blots for Fig. 6B.

**Additional file 5 **: **Fig. S5**. Full-length Western-blots for Fig. 7B.

**Additional file 6 **: **Fig. S6**. Full-length Western-blots for Fig. 8B

**Additional file 7 **: **Fig. S7**. Full-length Western-blots for Fig. 8D.

## Data Availability

The datasets used and/or analysed during the current study are available from the corresponding author on reasonable request.

## Abbreviations

ANOVA: Analysis of variance
BSA: Bovine serum albumin
DMEM: Dulbecco’s modified Eagle’s medium
FBS: Foetal bovine serum
GFAP: Glial fibrillary acidic protein Glioblastoma
GBM: Glioblastoma stem cells (GSCs)
GLUT-1: Glucose transporter 1
H: Hypoxia
HIFs: Hypoxia-inducible factors
IF: Intermediate filament
miRNAs: microRNAs
N: Normoxia
PRC2: Polycomb Repressor Complex
PVDF: Polyvinylidene difluoride
RNU6: RNA U6
SDS: Sodium dodecyl sulphate
TMZ: Temozolomide
TBS-T: Tris-buffered saline with 0.1% Tween-20
VEGF: Vascular endothelial growth factor
VHL: Von Hippel-Lindau (VHL)
