# Supplementary material for: The genotypic and phenotypic impact of hypoxia microenvironment on glioblastoma cell lines
Source: BMC Cancer. 2021 Nov 19;21:1248. doi: 10.1186/s12885-021-08978-z (PMC8605580; doi:10.1186/s12885-021-08978-z)

**The genotypic and phenotypic impact of hypoxia microenvironment on glioblastoma cell lines**

**Lucy Wanjiku Macharia^1,2^, Wanjiru Muriithi ^2,3^, Carlos Pilotto Heming^2,3^, Dennis Kirii Nyaga^2,4^, Veronica Aran^2^, Marianne Wanjiru Mureithi^5^, Valeria Pereira Ferrer^2^, Attilio Pane^3^, Paulo Niemeyer Filho^2^ and Vivaldo Moura-Neto^1,2*^**

**Additional file 1: Fig. S1 Expression of the KI-67 under different microenvironments.**


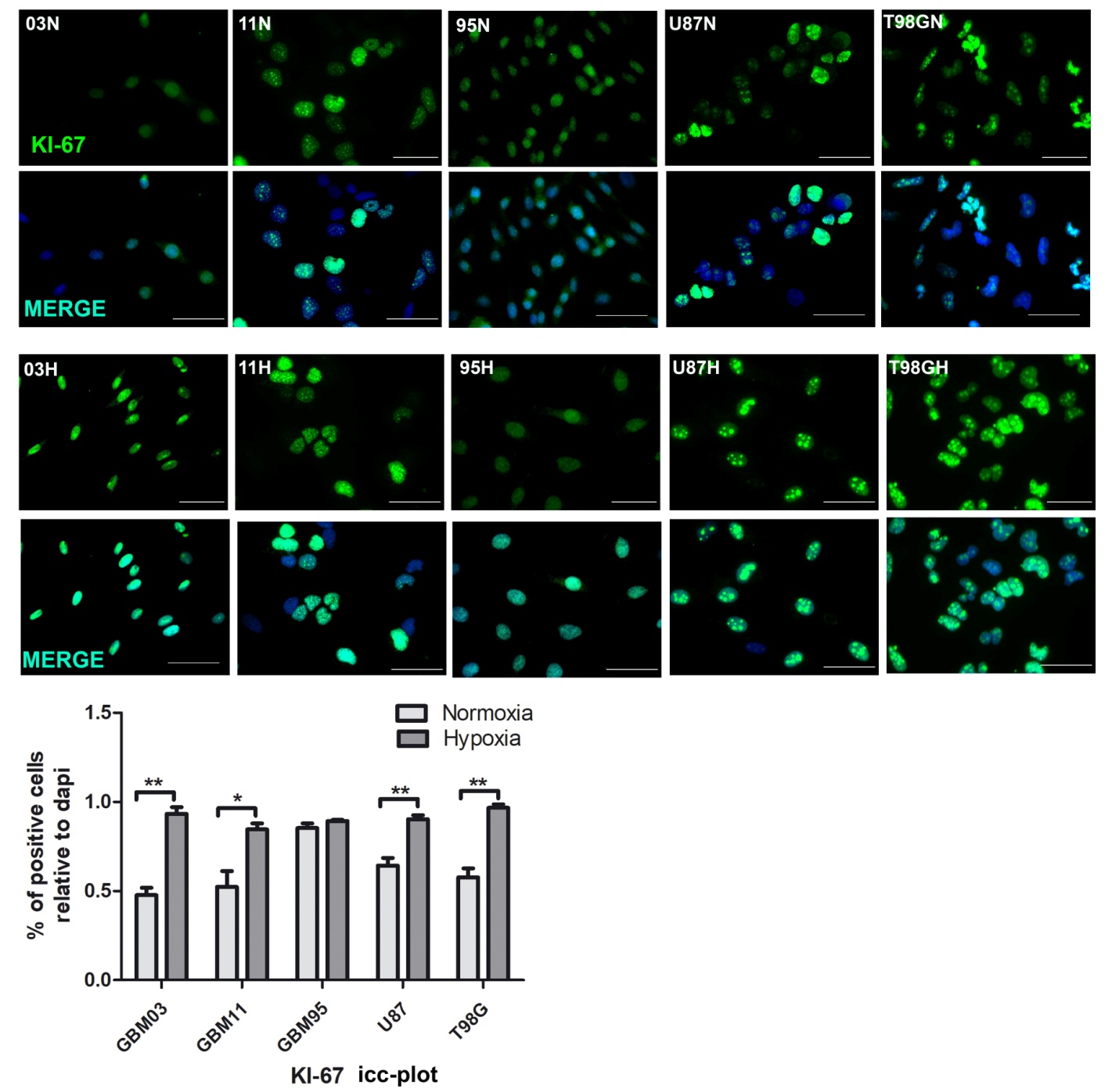

Supplement: Supplementary file 1 — Additional file 1 Expression of the marker of proliferation under different microenvironments. A. Cells were cultured under normoxia (N) and another set in hypoxia (H) for 72 h and stained for KI67. The Images were taken with DMi8 Leica microscope and prepared and quantified using ImageJ. Western blotting was done by the SDS-PAGE method and densitometry done using ImageJ. The relative expression was normalized to tubulin. The graphs were drawn and analysed using t test by graph pad prism. Each value represents the mean ± SD of three independent experiments, * indicates p < 0.05. Scale bar = 50 μm. [file 12885_2021_8978_MOESM1_ESM.docx]
