# Supplementary figures and images for: The genotypic and phenotypic impact of hypoxia microenvironment on glioblastoma cell lines
Source: BMC Cancer. 2021 Nov 19;21:1248. doi: 10.1186/s12885-021-08978-z (PMC8605580; doi:10.1186/s12885-021-08978-z)

**Additional file 2: Fig. S2 Full-length Western-blots for figure 4C**


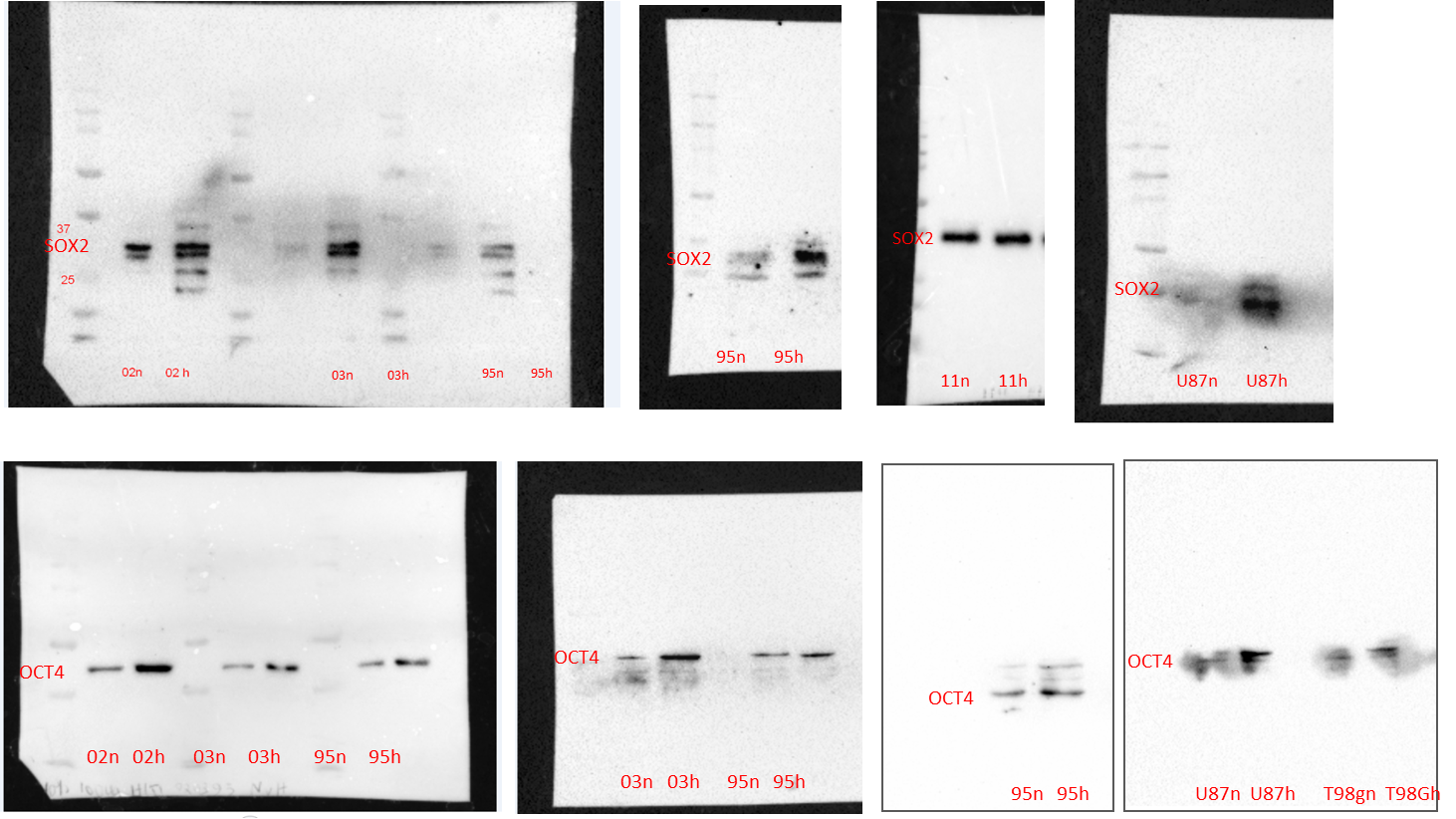

Supplement: Supplementary file 2 — Additional file 2 : Fig. S2. Full-length Western-blots for Fig. 4C. [file 12885_2021_8978_MOESM2_ESM.docx]

**Additional file 3: Fig. S3 Full-length Western-blots for figure 5B**

**
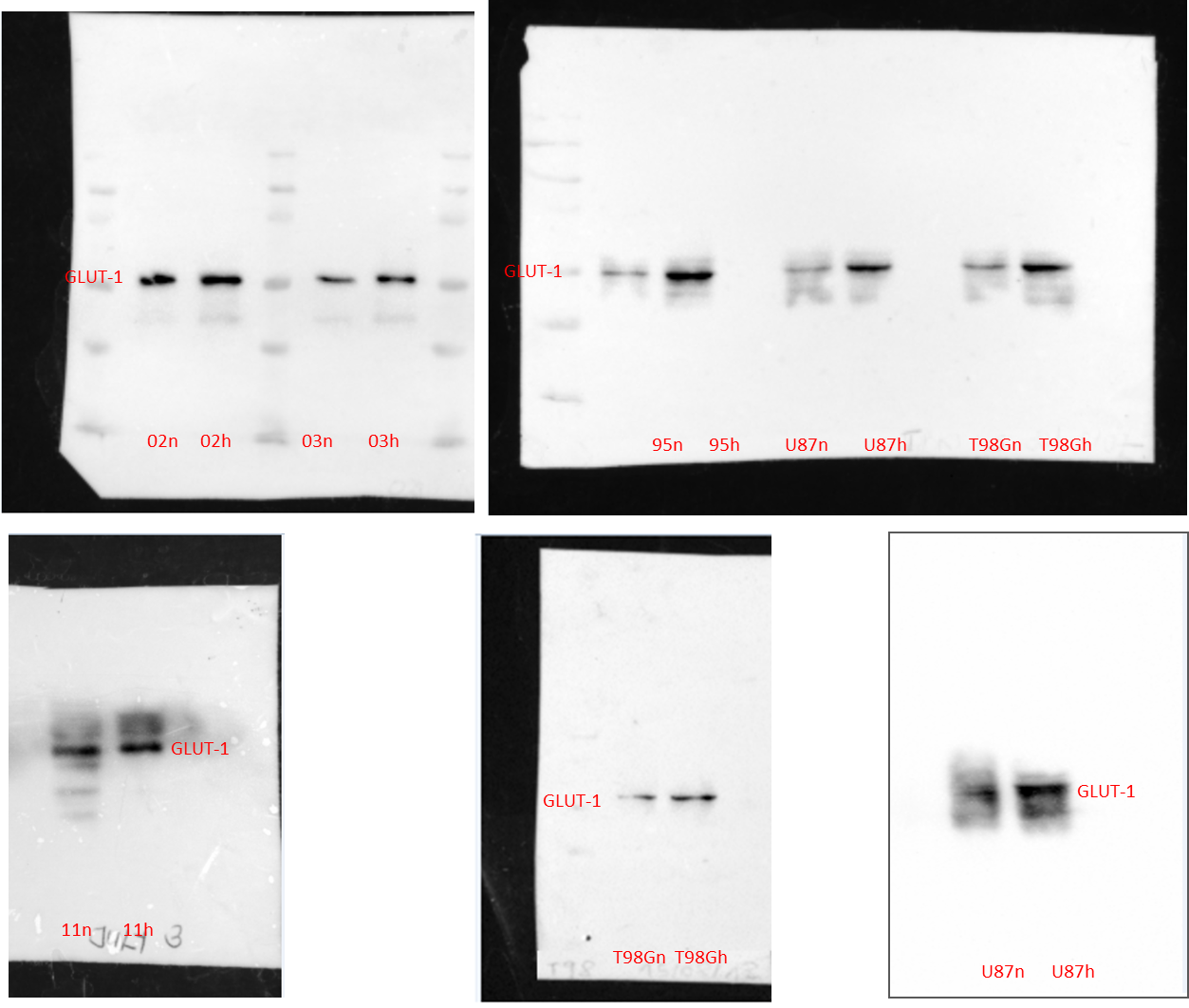
**

Supplement: Supplementary file 3 — Additional file 3 : Fig. S3. Full-length Western-blots for Fig. 5B. [file 12885_2021_8978_MOESM3_ESM.docx]

**Additional file 4: Fig. S4 Full-length Western-blots for figure 6B**

**
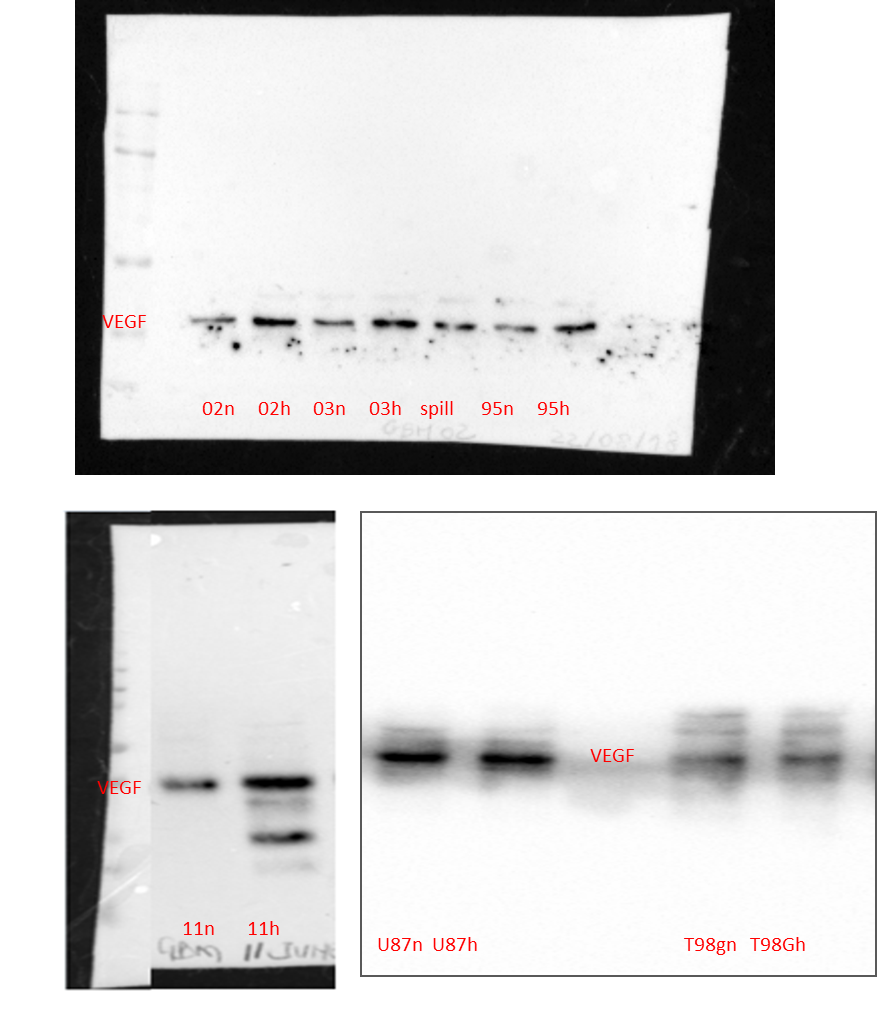
**

Supplement: Supplementary file 4 — Additional file 4 : Fig. S4. Full-length Western-blots for Fig. 6B. [file 12885_2021_8978_MOESM4_ESM.docx]

**Additional file 5: Fig. S5 Full-length Western-blots for figure 7B**


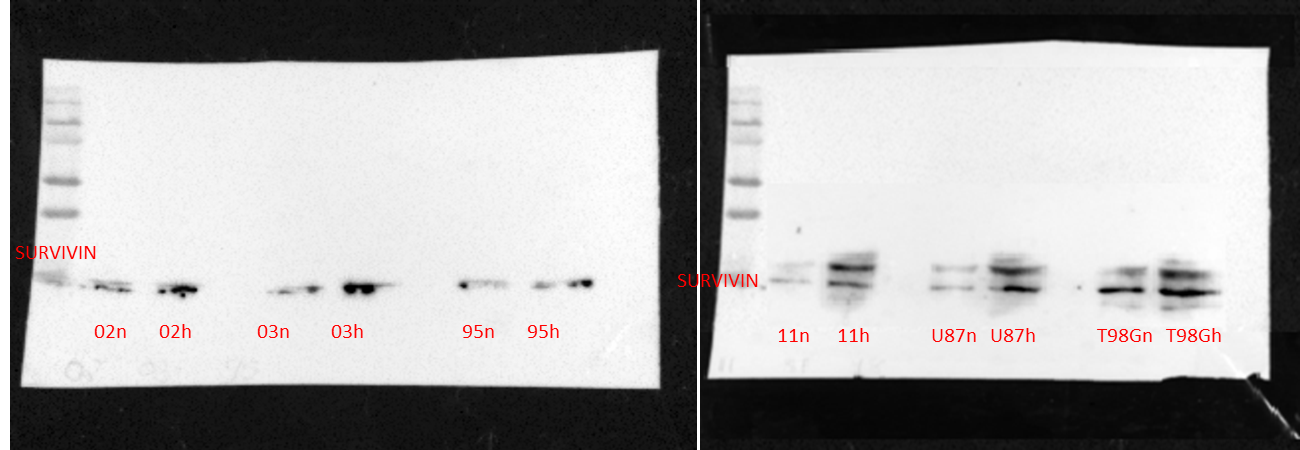

Supplement: Supplementary file 5 — Additional file 5 : Fig. S5. Full-length Western-blots for Fig. 7B. [file 12885_2021_8978_MOESM5_ESM.docx]

**Additional file 6: Fig. S6 Full-length Western-blots for figure 8B**


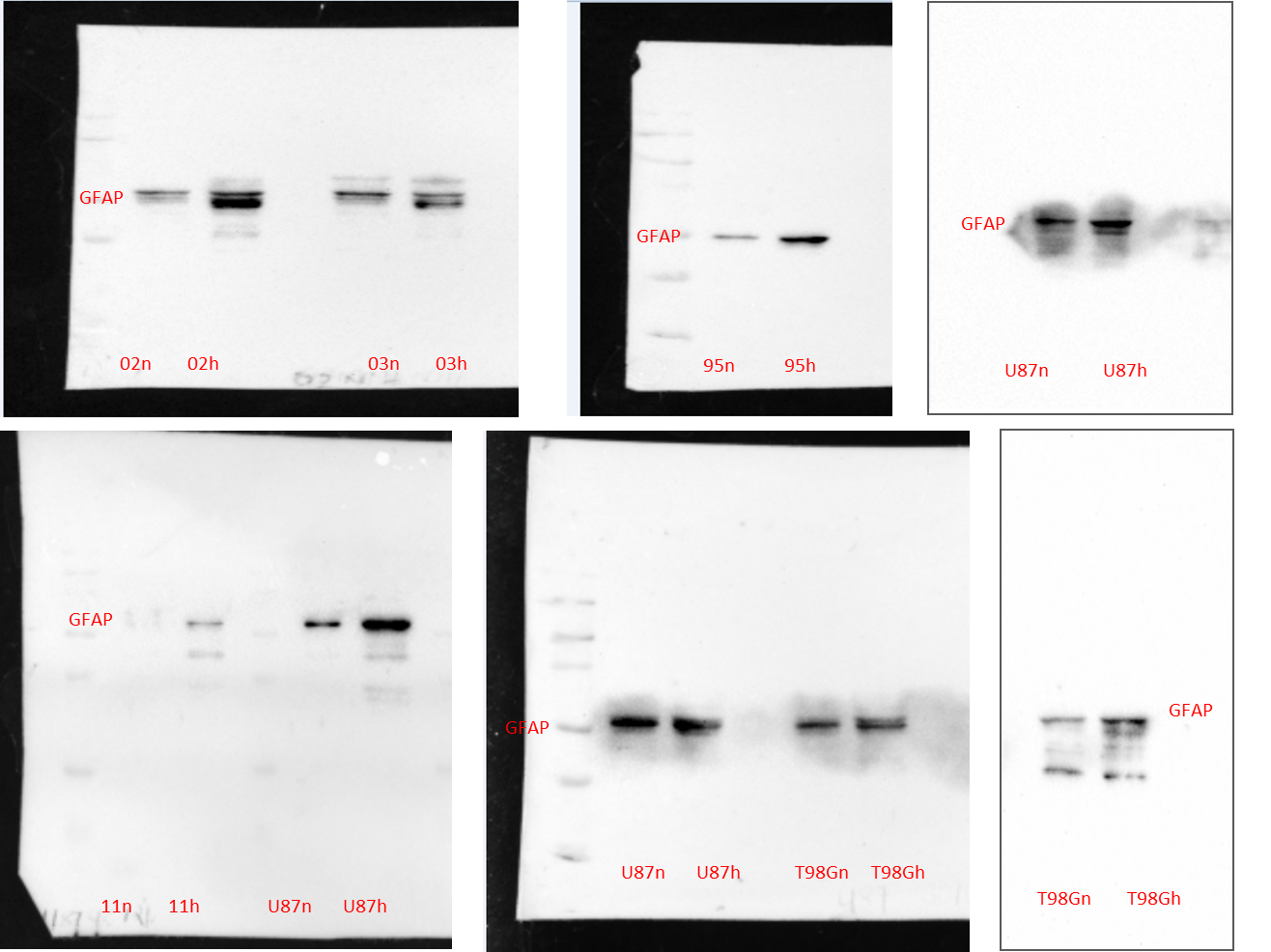

Supplement: Supplementary file 6 — Additional file 6 : Fig. S6. Full-length Western-blots for Fig. 8B [file 12885_2021_8978_MOESM6_ESM.docx]

**Additional file 7: Fig. S7 Full-length Western-blots for figure 8D**


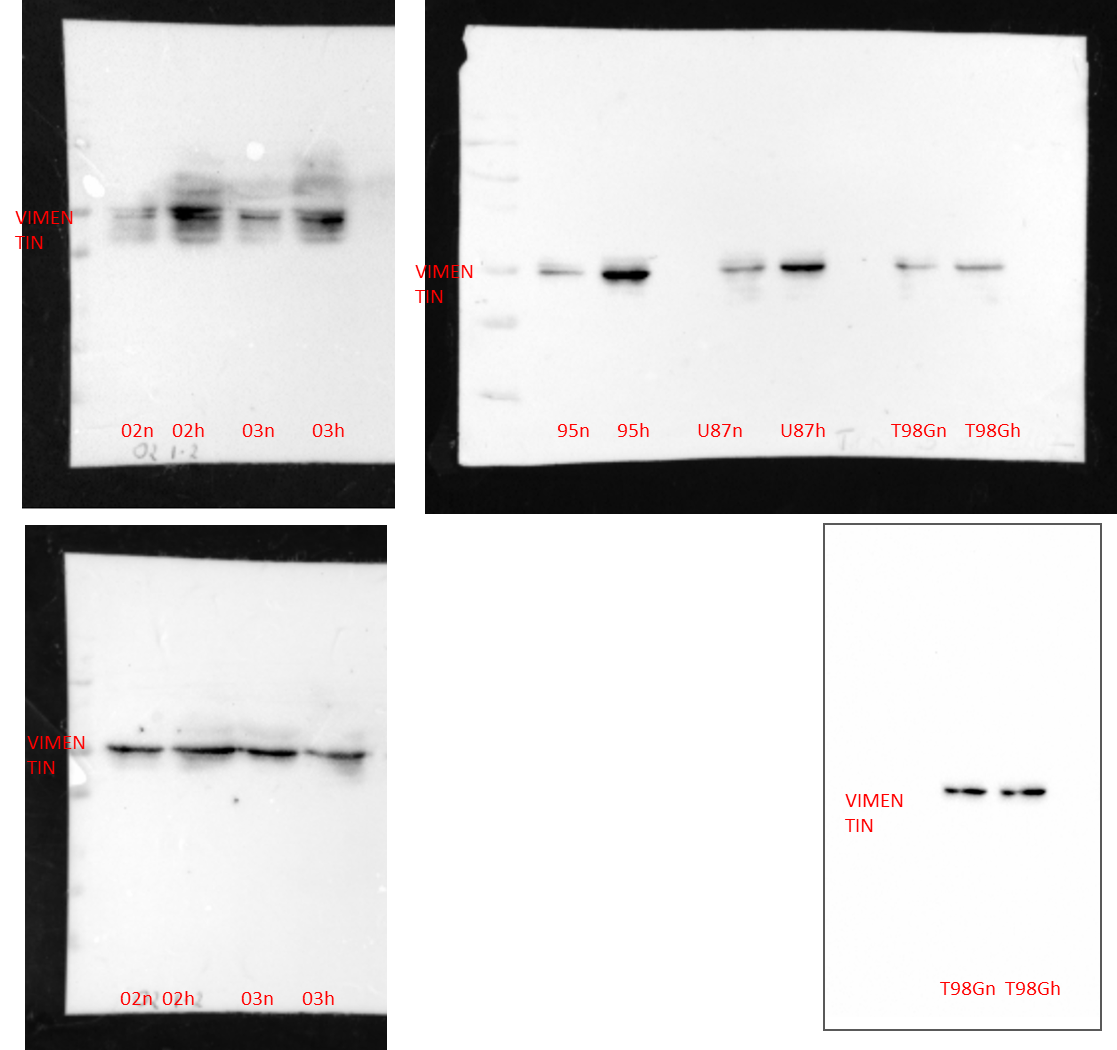

Supplement: Supplementary file 7 — Additional file 7 : Fig. S7. Full-length Western-blots for Fig. 8D. [file 12885_2021_8978_MOESM7_ESM.docx]
